# Simulation and verification of a hybrid ferry propulsion system for maritime operation in Saudi Arabia

**DOI:** 10.1038/s41598-026-53157-4

**Published:** 2026-05-13

**Authors:** Hussam A. Banawi, Mohammed O. Bahabri, Fahd A. Hariri, Mohammed N. Ajour

**Affiliations:** Department of Electrical and Computer Engineering, Faculty of Engineering, King Abdulaziz University, Jeddah, 21589 Saudi Arabia

**Keywords:** Marine, Propulsion, Energy management system, SRPM, Modeling, Energy science and technology, Engineering

## Abstract

Hybrid marine propulsion systems are becoming increasingly indispensable to support the analysis of energy management performance and system-level control behavior, especially of small vessels under variable load conditions. Nevertheless, a significant percentage of existing simulation research is based on simplified operational profiles or subsystem-level validations and thus limits their applicability to realistic maritime environments and full energy management assessments. The present investigation is aimed at developing and evaluating a modular hybrid propulsion system model, which is able to effectively synchronize the propulsion dynamics, power distribution, and energy storage performance under controlled simulation conditions representative of typical maritime operating profiles. A comprehensive hybrid power system is formulated and simulated in MATLAB/Simulink. It consists of a synchronous reluctance assisted permanent magnet generator (SRPM), a lithium-ion battery, and an SRPM propulsion motor. A hierarchical energy management architecture, based on state of charge (SOC) droop control and proportional-integral (PI) current control, is used to coordinate generator-battery power sharing and maintain DC-bus stability during dynamic operating situations. Simulation results indicate consistent system-level behavior under the considered operating conditions of the model in terms of propulsion speed tracking error of about 0.7%, torque tracking error of about 2%, DC-link voltage transient deviations of + 4.9% and − 3.6% with tighter regulation during steady-state operation, d-q axis current tracking error of 1% and 2%, total power tracking error of 3%, and battery SOC tracking errors of about ± 1% within the safe operating envelope of battery SOC of 20–80%. The proposed framework is a scalable and forward-compatible simulation platform for hybrid marine propulsion analysis. It enables the assessment of energy management strategies and opens the door for the integration of hydrogen fuel cells and renewable energy sources in the future to support the development and system-level evaluation of hybrid maritime propulsion systems aligned with Saudi Arabia’s Vision 2030.

## Introduction

### Literature review

The international shipping industry is one of the main sources of global GHG emissions, contributing almost 3% to the world’s total CO₂ emissions. In recognition of this challenge, the International Maritime Organization (IMO) has promulgated more stringent regulatory measures, such as the Energy Efficiency Existing Ship Index (EEXI) and the Carbon Intensity Indicator (CII), to promote decarbonization and boost energy efficiency across the sector^1^. Concurrently, Saudi Arabia has, through Vision 2030 and ambitious green hydrogen development programs, including NEOM, launched national programs to reduce emissions in the maritime sector and promote clean transport to further solidify its position as a global trade center^2^. These developments have led to an increasing interest in advanced energy savings and emission reduction technologies for ship propulsion by scholars and industries both on the international and regional levels, with hybrid and renewable energy technologies being seen as key enablers for sustainable maritime operations^3,4^.

The global movement towards renewable energy has turned into a high-profile, worldwide commitment, driven by regulatory demands, as well as social pressure to decrease GHG emissions^5,6^. Wind, battery storage and hydrogen fuel cells are some of the rapidly emerging technologies that would be the backbone of the future propulsion systems^7,8^. Initial studies of the modeling of hybrid electric ships and an initial energy management system (EMS) control have provided the basis of power split optimization and SOC management. On the international level, EU funded projects have augmented IMO decarbonization targets by certifying large scale clean energy incorporation in the corporate shipping industry. The FLAGSHIPS Project^9^ demonstrated the feasibility of hydrogen powered ships in clean waterborne transport across Europe and the MARANDA Project^10^ demonstrated a complete powertrain of a marine fuel cell in the harsh Arctic environment. These projects assist in contributing to the technical maturity of hydrogen and battery systems and offer operating data and policy guidelines to inform the future design of hybrid propulsion systems^11,12^. The stable nature and flexibility of the methodology have also made MATLAB/Simulink be used extensively in the research of naval power, enabling the detailed simulation of the system behavior, energy management and control strategies in a variety of load conditions.

Nevertheless, myriad existing models tend to oversimplify realistic maritime operating contexts—including the stochasticity of dynamics, external perturbations, and variable load profiles—thereby constraining the direct translatability of their findings to operational marine environments. Consequently, in spite of such endeavors, a small number of studies present a comprehensive simulation-based modular model in the form of a MATLAB/Simulink model that achieves representative component-level modeling, supports representative system-level simulation, and provides a modular structure compatible with the future integration of emerging renewable technologies, a limitation that the current study aims to partially address.

Park et al.^13^ proposed a real-time model-predictive control strategy for shipboard power management based on the IPA-SQP algorithm, and the control achieved rapid and optimal power dispatch under dynamic operating conditions; however, the control strategy did not consider an exhaustive multi-source hybrid propulsion model and component-level maritime verification. Torreglosa et al.^14^ showed real-time hybrid ship modelling in MATLAB/Simulink with the Hardware-in-the-Loop (HIL) techniques to capture the transient dynamics and energy flow control, but the proposed framework was not modular for incorporating future power sources. Zhang et al.^15^ took a step forward in shaft-speed control through an adaptive Proportional Integral Derivative (PID) control, but their treatment was still limited to propulsion dynamics without system-level coupling. Tang and Wang^16^ included realistic ramp rate and load-dependent dispatch rules for energy management but did not validate with physical components and did not fully integrate the dynamics of the battery and motion, limiting its applicability to the complete system. Wang et al.^17^ synthesized zero-carbon shipping strategies that spanned short- to long-term options: alternative low-carbon fuels, power system optimization, and onboard carbon capture, linking them to IMO targets; however, the work was limited to a system planning and policy level without verified mathematical modelling language (MATLAB/Simulink) or high-level simulation HIL testing, which limits its value for component-level simulation or controller design. Bassam^18^ modelled hybrid power performance over ferry routes based on real data, but its single scenario design prevents wider use. Chen et al.^19^ created a degradation-aware battery model with temperature and electrochemical effects, but the verification was only self-assessment, and there was no system-level testing. Hein^20^ suggested multi-objective energy and power regulation by integrated control loops, but important driving factors were not considered, and no real-world verification was given. Tuninetti et al.^21^ used hybrid power load data to design a propeller shaft, which improved the mechanical stress analysis but did not consider the electrical and control aspects. Araujo et al.^22^ provided a macro-level perspective on energy diversification for regional planning without insights on simulation or control. Nielsen et al.^23^ noted low-detail ship models that underestimated transient energy use, emphasizing component-level fidelity but with no complete control system integration for real-time applications. Wu et al.^24^ modelled tugboat maneuvering to study motion control in tight ports, providing knowledge of transient load cases but not energy use modelling. Radica et al.^25^ compared series/parallel/combined hybrid architectures but were based on conceptual designs with no real ship verification. Khooban et al.^26^ used fuzzy logic for DC-DC converters for better voltage regulation but did not consider integration with full motor or energy management loops. Cha et al.^27^ evaluated the model predictive and dynamic programming controls for electric boats, but most of the cases assumed full fuel cell adoption and were not very relevant to current diesel-battery hybrids. Jung et al.^28^ modelled hybrid power operations with energy use and emissions, but the framework was not flexible enough to be widely applicable. Andersen^29^ constructed a time domain model incorporating energy flow and control interactions, but this relied on ideal data and had no real-world verification. Inal et al.^30^ presented a critical review of mixed naval power systems in terms of their adaptability and control characteristics as well as the absence of a unified design operational platform. Geertsma et al.^31^ proposed concepts of fault-tolerant control of “smart” hybrid propulsion, but their methods were mostly theoretical. Balsamo et al.^32^ optimized the battery supercapacitor sizing using multi-objective techniques, but their analysis considered only storage and not the design of the entire system. Bennabi et al.^33^ emphasized mixed power of small vessels due to weight and space limits, which is a realistic factor that is usually absent in purely theoretical models, but the authors did not discuss energy management or system-level integration. Tang et al.^34^ suggested prognostics and health management (PHM) to naval energy management, which allows control changes based on the health of the component, and thus enhances reliability, but no experiment was conducted using a fully closed motor loop under a complex sea load. Hinic et al.^35^ confirmed a mixed propulsion model using real operating data, which gives information on energy saving and performance, but the model structure was inflexible to add new energy sources. Unlubayir et al.^36^ compared energy management techniques of battery solid-oxide fuel cell systems on bench tests and Simulink models, which provided reasonable results with diesel-battery hybrids and limited design flexibility. Arish et al.^37^ surveyed advances in electric boat propulsion, including permanent magnet motors, high-voltage transformers and built-in batteries, but said little about modeling or control system design. Joseph et al.^38^ proposed an optimized electric propulsion system using matching motor size to the actual load demand and demonstrated in Simulink how tuning of inverters influences the quality of propulsion but omitted the use of batteries and generators. Guo et al.^39^ compared centralized, decentralized and distributed EMS architectures and described the fault tolerance and SOC control but not tested models. Kyaw^40^ compared diesel, electric and hybrid propulsion in the presence of tactical stress and showed fuel savings in the variations of speed, the model was however simplified and lacked the dynamic coupling of battery and generator behavior.

Numerous studies have advanced hybrid marine propulsion modeling using MATLAB/Simulink, exploring aspects such as real-time and HIL simulations for transient dynamics and energy flow strategies^13–16^; advanced energy management and control methods^19–22^; and component-level optimization including battery aging, predictive maintenance, and storage sizing^41–44^. Collectively, these works have deepened subsystem understanding, yet most remain limited to simplified scenarios, lack component-level accuracy under controlled simulation conditions, and seldom employ modular architectures capable of integrating future renewable sources. Consequently, few provide a comprehensive and adaptable framework evaluated through simulation, required when many domains interact under changing marine environments. In light of these limitations, this study develops an integrated, modular, and forward-compatible MATLAB/Simulink framework to address them.

In addition to component-level and system-level modeling efforts, various energy management strategies have been explored in hybrid marine propulsion and microgrid applications, including rule-based control, optimization-based methods such as ECMS and MPC, and more recently, data-driven approaches such as reinforcement learning^16,27,39,43^. Rule-based strategies are generally simple and suitable for real-time implementation but may lack adaptability under highly dynamic operating conditions. Optimization-based approaches can achieve improved energy efficiency; however, they typically rely on accurate system models and involve higher computational effort. Meng et al.^43^ has suggested an energy management approach of online reinforcement learning, which is based on SARSA algorithm in a centralized wind-photovoltaic-energy storage microgrid. The approach allowed adaptive decision-making in the presence of uncertainty and had lower computational cost compared to some traditional optimization-based and deep learning-based counterparts. However, the model was designed to schedule energy in microgrids and not to propel a marine vessel and lacked dynamics of a propulsion-system and a combined generator-battery control, which restricts its direct use in hybrid ship propulsion research. The adaptive abilities of reinforcement learning-based approaches provide them with capabilities to operate in uncertain conditions, although their application is frequently restricted by the training needs and computational complexity. However, the EMS strategies are discussed based on literature-reported characteristics and conceptual comparison are not implemented or evaluated under identical simulation conditions within this study. Therefore, the comparison remains qualitative and is intended to position the proposed approach rather than provide a direct performance benchmark.

The proposed SOC-based droop strategy is an algorithm that is tailored to explicitly incorporate energy management with DC-link regulation and propulsion dynamics into a single control framework, unlike optimization-based EMS like MPC and ECMS^16,27^ that use predictive models and computationally intensive optimization routines. This allows real-time power coordination without predicting future loads or model complexity of the system. Unlike the reinforcement learning methods^43^, which rely on extensive training and data access, the method suggested offers deterministic and consistent performance in the face of changing operating conditions, thus, being more applicable to the practical marine application where computational simplicity and robustness are paramount. This design allows direct interaction between energy management decisions and system-level electrical and propulsion dynamics, which is not commonly addressed in many existing EMS implementations for marine propulsion systems.

To offer a systematic comparison of the available energy management and control schemes in hybrid marine propulsion systems, Table 1 presents the summary of representative studies reported in the literature according to their methodology, performance features, and limitations.

**Table 1:** Comparative analysis of representative energy management and control strategies in hybrid marine propulsion systems.

Ref	System/application	EMS/control method	Marine suitability	Key reported contribution	Main limitation	Comparative characteristics
^13^	Hybrid ship power system	MPC (IPA-SQP)	Medium	Fast dynamic response and optimized power dispatch	High computational complexity and model dependence	Suitable for real-time implementation with lower computational requirements
^14^	Hybrid ship MATLAB/Simulink model	Real-time modeling with HIL-based energy-flow control	High	Captured transient dynamics and energy-flow behavior in real time	Limited modularity for integrating additional energy sources	Modular and extensible system architecture
^15^	Propulsion shaft-speed control	Adaptive PID control	High	Improved propulsion shaft-speed regulation	Focus limited to propulsion dynamics without full system integration	Includes coordinated control of propulsion, generator, battery, and DC-link
^16^	Hybrid marine EMS	ECMS/rule-based dispatch	Medium	Near-optimal fuel-saving potential with load-dependent dispatch	Requires accurate system modeling and limited system-level integration	Reduced model dependency and simplified implementation
^18^	Ferry hybrid power system	Route-based performance modeling	High	Evaluated system performance using real ferry-route data	Limited generalizability to different operating conditions	Flexible and generalizable simulation framework
^20^	Marine hybrid energy system	Multi-objective control loops	Medium	Coordinated energy and power regulation strategy	Some operating factors not fully considered and limited validation	Comprehensive system-level coordination
^25^	Hybrid propulsion architectures	Architecture comparison (series/parallel)	High	Comparative analysis of hybrid propulsion configurations	Conceptual-level analysis without dynamic system validation	Provides dynamic system-level simulation
^26^	Converter-level subsystem	Fuzzy-logic DC-DC converter control	Medium	Improved voltage regulation at converter level	Limited integration with full propulsion and EMS system	Designed for integrated propulsion and energy management coordination
^27^	Electric boat propulsion	MPC/DP-based EMS	Medium	Improved energy efficiency under predefined cycles	High computational demand and limited real-time feasibility	Suitable for real-time embedded implementation
^28^	Hybrid power system with emissions analysis	Energy and emissions modeling	High	Considered energy consumption and emissions impact	Limited flexibility for different system configurations	Adaptable modular framework
^29^	Hybrid propulsion dynamic model	Energy-flow and control-interaction modeling	High	Modeled energy flow and control interactions over time	Based on idealized conditions without experimental validation	Suitable for control-oriented integrated analysis
^31^	Hybrid propulsion architectures	Rule-based supervisory control	High	Stable operation with low computational complexity	Limited adaptability and mainly conceptual validation	Adaptive SOC-based droop control within integrated framework
^32^	Hybrid energy storage system	Multi-objective optimization	Medium	Improved storage sizing and energy efficiency	Focus on storage subsystem rather than full propulsion integration	Full system-level integration
^34^	Naval hybrid energy system	PHM-assisted EMS	High	Adapted control based on component health conditions	Limited validation under full dynamic propulsion conditions	Includes coordinated closed-loop system interaction
^35^	Hybrid propulsion with real data	Verified system modeling	High	Demonstrated performance using real operational data	Limited flexibility for adding new energy sources	Extensible architecture for future integration
^36^	Battery–fuel-cell hybrid system	Optimization-based EMS	Medium	Improved energy distribution and comparative EMS evaluation	Design-specific and limited flexibility	Generalizable modular framework
^39^	Hybrid propulsion EMS architectures	Centralized/decentralized EMS	Medium	Improved coordination and fault tolerance concepts	Increased system complexity and limited integrated validation	Simpler and more practical coordination strategy
^40^	Hybrid propulsion under varying conditions	Comparative propulsion evaluation	High	Demonstrated fuel savings under speed variations	Simplified modeling of subsystem interactions	Explicit dynamic coordination between generator and battery
^41^	PMSM propulsion system	Nonlinear propulsion control	High	High tracking performance with low overshoot	High control complexity	Comparable performance using simpler control strategy
^43^	Wind-PV-energy storage microgrid with centralized control	Online reinforcement learning-based EMS (SARSA)	Low	Adaptive energy management under uncertainty with improved computational efficiency relative to MPC and DQN	Developed for microgrid scheduling rather than marine propulsion systems	Proposed method is marine-specific and simpler to integrate within propulsion-oriented system-level control
This work	500 kW hybrid ferry propulsion system	SOC-droop EMS with coordinated PI control	High	Integrated propulsion control, power sharing, DC-link regulation, and SOC management with stable performance	Simulation-based validation only	Real-time feasible, low-complexity, and fully integrated system-level framework

Based on the comparison presented in Table 1, it can be observed that most existing studies focus on either subsystem-level optimization, propulsion-only control, or conceptual system architectures, with limited emphasis on integrated system-level simulation under representative marine operating conditions. These constraints underscore the importance of an integrated and versatile framework that can effectively deal with propulsion dynamics, energy management and power coordination.

According to the review provided above, it is possible to identify three major research gaps in the existing literature. To begin with, numerous works are conducted on the subsystem-level modeling, like propulsion motor control, energy management algorithms, or battery optimization, without incorporating these aspects into a single hybrid propulsion architecture. Second, several works use simplified operating conditions or idealized load models, and this restricts the analysis of synchronized system-level dynamics of propulsion speed and torque control, DC-link voltage regulation, and battery SOC control. Third, only a limited number of studies provide modular MATLAB/Simulink frameworks that can be readily extended to incorporate emerging maritime energy technologies such as hydrogen fuel cells or renewable-assisted propulsion systems.

To fill these gaps, the current study provides a complete modular 500 kW hybrid ferry propulsion model, which incorporates a diesel generator, lithium-ion battery storage, and an SRPM propulsion motor, which is coordinated by an SOC-based droop energy management strategy. The suggested framework allows assessing the propulsion dynamics, the generator-battery power sharing, the DC-link stability, and the SOC regulation under the conditions of the representative maritime operating conditions. This combined modeling and simulation-based assessment system offers a holistic simulation platform that is not limited to most of the past subsystem-based research. This study focuses on the development and validation of a modular and computationally efficient EMS framework tailored for maritime applications, with emphasis on system-level integration and real-time feasibility.

### Main work and contributions

The main contributions of this study can be summarized as follows:

1. Design and implementation of a system-level MATLAB/Simulink model of diesel generator dynamics, lithium-ion battery storage, SRPM propulsion motor, and power electronic converters into a single hybrid propulsion design.
2. Implementation of a hierarchical control framework combining SOC-droop energy management with PI–MTPA propulsion motor control to achieve coordinated regulation of propulsion torque, DC-link voltage stability, and generator–battery power sharing.
3. Quantitative assessment of the propulsion system under a representative maritime operating profile with dynamic speed control, torque response, generation power coordination and battery SOC control.
4. Comparative qualitative discussion with representative hybrid marine propulsion studies to position the proposed control architecture in terms of dynamic performance and implementation characteristics.

Compared with many previously reported hybrid marine propulsion studies, the proposed system differs in three main technical aspects. First, regarding control architecture, the model incorporates a hierarchical control structure that is a combination of SOC-based droop energy control and PI-MTPA propulsion motor control that allows coordinated control of propulsion torque, DC-link voltage stability, and generator-battery power sharing. Second, the proposed design will consist of a SRPM propulsion motor, a lithium-ion battery storage and a diesel-powered generator on the component choice, which is connected through bidirectional power electronic converters to a single DC bus architecture. Third, regarding system integration, the overall propulsion system is deployed as a modular MATLAB/Simulink platform that allows simulating propulsion dynamics, energy management, and electrical interactions simultaneously, but can be expanded to include future systems integration of emerging energy sources like hydrogen fuel cells or renewable-assisted propulsion. These features distinguish the proposed model from many existing subsystem-oriented or non-modular hybrid propulsion simulations reported in the literature. The contribution of this work is incremental rather than conceptual, focusing on the coordinated integration of propulsion dynamics, SOC-droop energy management, and DC-link regulation within a unified modular simulation framework under representative operating conditions.

Though many of the individual elements of hybrid marine propulsion, including diesel generators, lithium-ion batteries, permanent magnet machines, PI current control, and droop-based power sharing have been previously studied in isolation, the originality of this study is that these elements have been combined in a coordinated manner in a single and modular system-level simulation framework. Contrary to several of the previous works, which consider isolated subsystems or simplified operational conditions, the proposed model allows to examine the propulsion dynamics, generator-battery power coordination, DC-link voltage stability, and SOC regulation under ferry operating conditions. This level of system integration offers an end-to-end platform to assess the interactions between propulsion control, energy management and electrical power distribution, and has a modular architecture to enable future integration of new maritime energy technologies, including hydrogen fuel cells or renewable-assisted propulsion.

A reliable hybrid propulsion model that can be developed must be much more complicated than just replacing a diesel engine with some other form of propulsion modality. Maritime operations are nonlinear and dynamic and are full of numerous uncertainties. In order to provide scientific rigor before the findings can be implemented in the practice, in the given study, the detailed, modular, mathematical MATLAB/Simulink model of a 500-kW hybrid ferry that works under the conditions of the representative and simulation-based maritime environment, which represents a controlled simulation scenario inspired by typical operating conditions in Saudi maritime environments. The model combines a diesel generator and a lithium-ion battery and explicitly represents the load behavior, interactions between the battery and the generator responses under controlled simulation conditions with data in the archival or literature-based. The transparent and modular design will offer forward compatibility and ability to add new renewable resources and advanced control algorithms in the future, in effect giving the model the technical capability and flexibility its needs in the long term to be able to survive. The proposed EMS enables stable power flow, improves generator load sharing and reduces transient loading and maintains optimal SOC under various load profiles. The model in this study is more effective in describing the coordinated system-level interactions in the controlled simulation environment than previous studies because it takes into account the transient load variations, control interactions, and complex interactions between the components. Subsequent development stages may add hydrogen fuel cells and wind turbines without requiring wholesale redesign of the existing framework, and the model is therefore in a good position for future energy conversion technologies.

The present study focuses on system-level simulation of the proposed hybrid propulsion architecture using a MATLAB/Simulink environment. The model is evaluated under controlled simulation assumptions, including ideal sensor measurements, negligible actuator delays, and the absence of explicit hydrodynamic disturbances such as wave-induced loads and wind effects. These assumptions are intentionally adopted to isolate and evaluate the performance of the proposed energy management and control framework. Experimental validation using hardware-in-the-loop platforms or full-scale onboard testing is considered an important direction for future research. It should be noted that the present study focuses on system-level dynamic performance and control behavior of the hybrid propulsion system. Detailed evaluation of fuel consumption, system efficiency, and emissions reduction is not explicitly included in the current simulation framework, as such analyses require high-fidelity component models and experimentally validated fuel and emission characteristics. These aspects are considered as important directions for future work.

The remainder of the paper is organized as follows: Sect. 2 gives an overview of the main components of the model and how they were developed. Section 3 introduces the propulsion system, battery, and diesel generator control logic. Section 4 presents simulation scenarios and results in detail, showing the stability and adaptability of the model. Section 5 closes with a summary of important findings, an explicit statement of research limitations (e.g., the need for longer-term multi-situation experimental verification), and specific future research directions.

**Fig. 1:**
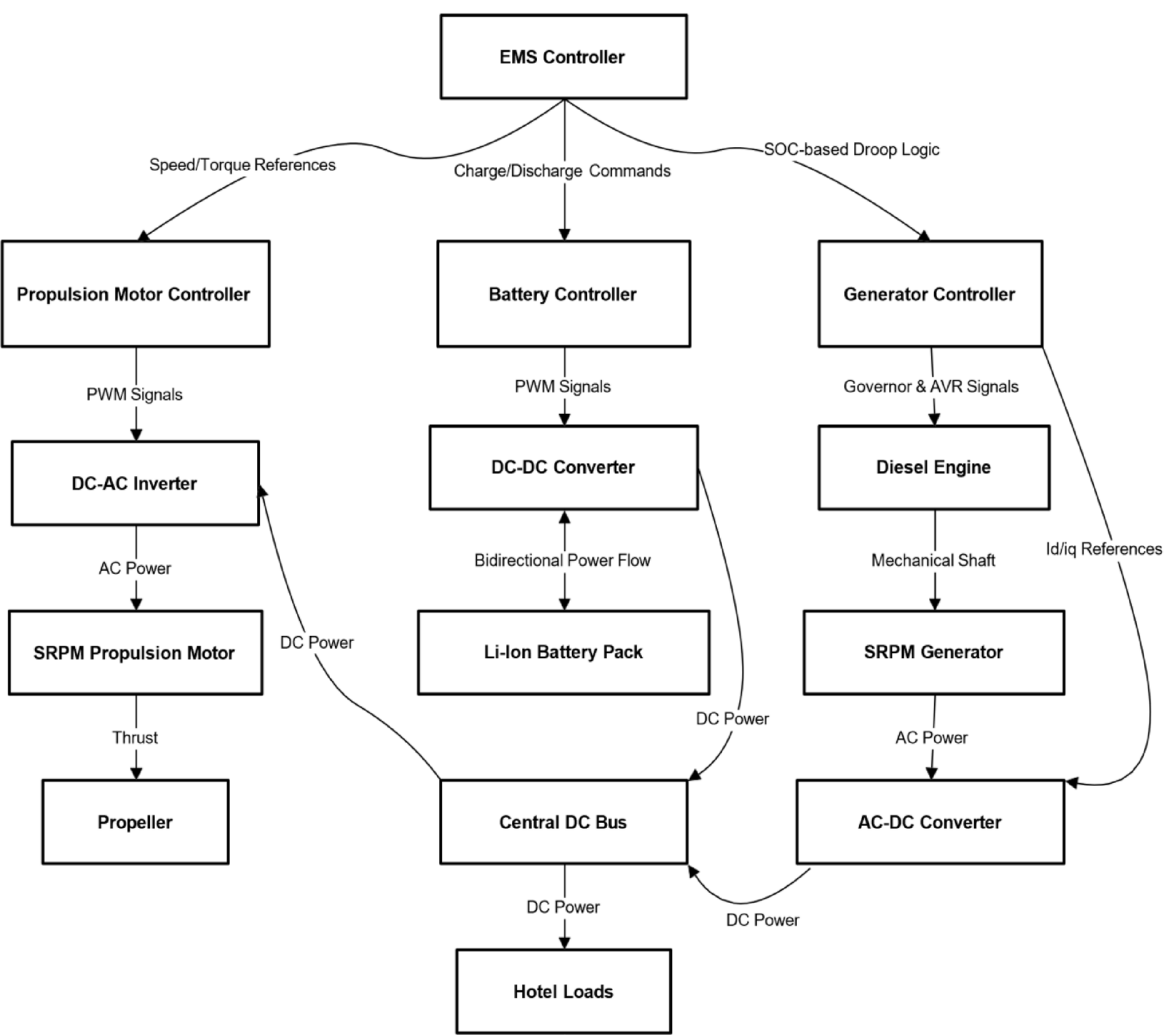
Overall energy management and power flow architecture of the hybrid propulsion system.

Figure 1 shows the general control and power flow structure of the proposed hybrid propulsion model. At the heart of the EMS controller is the coordination between the generator controller and the battery controller using SOC-based droop logic and charge/discharge commands. The diesel engine is connected to an SRPM generator, the output of which is supplied through an AC/DC converter to the central DC bus. At the same time, a lithium-ion battery pack controlled by a bidirectional DC/DC converter exchanges power with the same DC bus for load leveling and peak shaving. The speed/torque references sent to the propulsion motor controller from the EMS are used to drive the SRPM propulsion motor through a DC–AC inverter to produce thrust. Hotel loads are fed from the central DC bus. The energy flows and interaction of each controller are clearly shown in this architecture, which highlights the modular design and facilitates the future integration of renewable sources such as hydrogen fuel cells or wind power.

## System component modeling

The system is based on the MATLAB/Simulink R2025a (MathWorks) simulation of typical maritime operating conditions for a 500-kW hybrid vessel that consists of a diesel generator and a lithium-ion battery. By shifting the transient load swings to the battery while the diesel generator maintains the continuous base load, the design improves power distribution and reduces transient loading on the generator. The architecture is also flexible and scalable in order to accommodate future integration of renewable sources such as wind or hydrogen fuel cells. Intelligent control algorithms are used to enable dynamic balance and responsiveness by power exchange between subsystems in simulated conditions. Each subsystem (converters and the propulsion motor) is modeled in a common simulation environment, which reproduces representative system-level behavior. All the component parameters are based on commercially available SRPM machines, lithium-ion batteries, and standard power converters and thus achieve a balance between realism and generalizability suitable for evaluating the proposed energy management and SOC droop control strategies.

### Battery modeling

The 500-kW ferry propulsion system 300-kW lithium-ion battery system was developed based on detailed operational analysis, technical specifications, and high-fidelity modeling. This capacity has been chosen with an analysis of a load profile of recorded ferry operation that indicated that there were transient peaks of propulsion and hotel loads that were higher than the base output of the diesel generator. Newer hybrid marine studies have used 50–70% sizing ratios of the primary propulsion power to provide sufficient transient support and SOC stability^25,32,36^. These momentary peaks had been set to the 300-kW operating point to make sure that the battery SOC did not go too low or too high so that the battery was not exposed to deep cycling and would have appropriate operational limits. Since the Saudi marine is harsh i.e. high ambient temperature, salinity with corrosive properties, a lithium iron phosphate (LFP) battery was chosen because of its high thermal performance, high power density, more than 10,000 charge cycles, low degradation at high temperature and high charging power. These characteristics are critical to marine reliability and durability. The battery model was parameterized with data (discharge curves) provided by the manufacturer in the MATLAB/Simulink environment to simulate the representative behavior of discharge under simulated operating conditions. In the case of system-level studies, the Rint model was employed because it has a good simplicity fidelity trade-off, where the battery is modeled as an open-circuit voltage (OCV) source with internal resistance given by Eq. (1)^19^:

1 $$\:{V}_{bat}={V}_{oc}-I\cdot\:{R}_{int}$$

where:

- V_bat_ is the terminal voltage,
- V_oc_ is the OCV (function of SOC),
- I is the discharge current,
- R_int_ is the internal resistance.

The standard Coulomb counting method to determine the SOC of the battery is the integral of the actual current of the battery with respect to time rather than its derivative, which is given by Eq. (2)^19^:

2 $$\:SOC\left(t\right)=SOC\left(0\right)-\frac{1}{{C}_{nom}}{\int\:}_{0}^{t}\:{I}_{bat}\left(\tau\:\right)d\tau\:$$

where:

- SOC(t) is the battery SOC at time t,
- C_nom_ is the nominal battery capacity in Ah,
- I_bat_ is the battery current (positive during discharge).

The adopted Rint equivalent circuit model is intended for system-level dynamic analysis and does not capture detailed electrochemical phenomena such as temperature-dependent behavior, aging mechanisms, or transient polarization effects. In particular, the impact of high transient current profiles on battery lifetime is not explicitly represented. Such effects require more advanced electrochemical or semi-empirical aging models coupled with long-term cycling data, which are beyond the scope of the present study. However, the chosen model is a good trade-off between the sophistication of the calculations and the quality of the assessment of the energy management and power coordination. The more realistic battery models, including thermal and aging, will be added to the future work to make the simulation as realistic as possible in maritime under operating conditions. Moreover, much literature has been done on the application of hybrid energy storage system with supercapacitors as a complementary measure to high frequency transients of power in dynamic energy systems. Such systems usually employ supercapacitors to store high power transient spikes to eliminate transient current stress on the battery and enhance the life of the overall system^44–46^. The current work is centered on a battery-based system to test system-level energy management and control performance under representative operating conditions. Future research involves the integration of supercapacitors and the evaluation of its effect on the transient reduction of current and battery life.

### Machine modeling

The reason why the SRPM machine was selected in this study is that it is very efficient, compact in design and has been well reported to be reliable in marine propulsion applications. Notably, the identical SRPM machine is utilized as the principal propulsion motor and as the generator that is linked to the diesel engine. This dual capability makes it especially well suited for hybrid electric marine architecture, which can efficiently generate and propel within a single integrated platform.

Among the models available, the EM-PMI375 series is the most efficient on the market, with a market leading efficiency of up to 96% and outstanding performance over a broad operating range. The selected variant, T1100, provides continuous torque of 1100 Nm and a power range of 177–296 kW, which is well suited to meet the propulsion and power generation needs of the ferry. The liquid-cooled aluminum motor housing provides thermal stability under long-term operation, and the IP67 ingress protection rating increases durability in corrosive marine environments. High-voltage operation and advanced control implementation are seamlessly integrated with the EC-C1700B inverter in the Simulink environment.

Since an SRPM model is not available in MATLAB/Simulink, a generic synchronous machine model is modified. The motor parameters, like saliency ratio, inductance profiles, torque characteristics, and efficiency curves, are adjusted to represent the real behavior of the SRPM unit. The machine model is based on the conventional dq-axis transformation approach for synchronous machines^37^, which allows the electromagnetic torque and flux calculations in both motoring and generating modes. The modified synchronous machine model with constant d- and q-axis inductances (Ld and Lq) representing the average magnetic properties of the SRPM is used in its linear operating region. This linear approximation is convenient to implement and provides numerical stability for control-based simulation. The current work concentrates on the validation of the energy management and control performance of the hybrid system rather than the detailed electromagnetic behavior; therefore, magnetic saturation and dq cross-coupling effects are not explicitly modeled at this stage. The chosen Ld/Lq saliency ratio and flux linkage values were chosen to agree with manufacturer steady-state data for the EM-PMI375 (T1100) unit, which provides realistic torque and efficiency responses under dynamic load changes. This model offers a reasonable trade-off between fidelity and computational efficiency, allowing the machine’s behavior to be reasonably represented under the considered operating conditions (propulsion and power generation) and providing a solid basis for control development, system analysis, and future integration with other energy sources.

Voltage Equations as shown (3,4) in the dq Reference Frame^37,41^:

3 $$\:{V}_{d}=\:{R}_{s}{I}_{d}+\:{L}_{d}\frac{d{i}_{d}}{dt}-\:\omega\:{L}_{q}{I}_{q}$$

4 $$\:{V}_{q}=\:{R}_{s}{I}_{q}+\frac{{L}_{q}d{i}_{q}}{dt}+\:\omega\:{L}_{d}{I}_{d}+\:\omega\:{\lambda\:}_{m}$$

where:

- V_d_,V_q_ are the stator voltages in the dq-axis.
- I_d_,I_q_ are the stator currents in the dq-axis.
- R_s_ is the stator resistance.
- L_d_,L_q_ are the d-axis and q-axis inductances.
- λ_m_ is the permanent magnet flux linkage.
- ω is the rotor speed.

Torque Equation

The electromagnetic torque produced by the propulsion motor is given as described in Eq. (5)^37,41^:

5 $$\:Te=\frac{3}{2}\:P\left({\lambda\:}_{m}Iq+\left({L}_{d}-{L}_{q}\right){I}_{d}{I}_{q}\right)\:$$

where:

- Te is the electromagnetic torque.
- P is the number of pole pairs.
- λm is the permanent magnet flux linkage.

This equation describes the torque generation in terms of the interaction between the stator currents, inductances, and magnetic flux.

Mechanical Dynamics of the Motor as described in Eq. (6)^37^:

6 $$\:J\frac{d\omega\:}{dt}=\:{T}_{e}-\:{T}_{L}-\:B\:\omega\:$$

where:

- J is the moment of inertia.
- T_L_ is the load torque.
- B is the friction coefficient.

Adjusting the implemented model of the synchronous motor in MATLAB with the equations and parameter values unique to SRPM, a realistic propulsion motor model is obtained to simulate ferry propulsion systems.

### Technical specifications of the main components

The hybrid electric propulsion system was modeled and implemented and evaluated using MATLAB/Simulink in order to accurately represent the electrical, mechanical, and control dynamics of the vessel. In order to ensure reproducibility and improve clarity, the principal components along with their corresponding parameters are summarized in four tables, which include the energy storage subsystem, electrical machines, control loops, and Maximum Torque Per Ampere (MTPA) dataset used in the torque optimization.

Table 2 shows a summary of the main electrical and operational parameters of the 300 kW LFP battery pack. The battery pack is connected to the 750 V DC link via a bidirectional DC-DC converter and is operated in a conservative SOC of 20% to 80% to avoid deep cycling and thus extend its service life. A Rint equivalent circuit model (OCV and internal resistance) was to provide reasonable SOC estimation and voltage response under different load conditions.

**Table 2:** Lithium-ion battery specifications.

Parameter	Value	Unit	Description
Rated power	300	kW	LFP pack
Operating SOC window	20–80	%	Protects from deep cycling, extends life
DC bus nominal voltage	750	V	Nominal DC link voltage
Nominal capacity (C_nom_)	400	Ah	Equivalent to 300-kWh total energy
Equivalent circuit model	Rint (OCV + internal resistance)	–	Used for SOC estimation
Thermal stability	High	–	Suited for marine ambient temperature and salinity

Table 3 shows the electrical and mechanical parameters of the SRPM machines used for propulsion and generation. The EM-PMI375 (T1100) configuration is common to both units, selected for high efficiency (approx. 96%), compactness, and well-documented reliability in marine propulsion applications.

Stator resistance (Rₛ), dq inductances (Ld and Lq), permanent magnet flux linkage (λPM), rotor inertia (J) and viscous friction (fv) are introduced in the dq reference frame to ensure realistic torque and flux calculations.

**Table 3:** SRPM generator and propulsion motor specifications.

Parameter	Propulsion PMA-SRM	Generator PMA-SRM	Unit
Stator resistance Rs	0.007	0.007	Ω
d-axis inductance Ld	0.0003	0.0003	H
q-axis inductance Lq	0.0002	0.0002	H
Pole pairs pp	8	8	–
PM flux linkage λPM	0.256	0.256	Wb
Rotor inertia J	4.73	4.73	kg·m^2^
Viscous friction fv	0	0	N·m·s
Electrical time constant	0	0	s
Initial speed	1000	1000	rpm
Initial electrical angle	0	0	rad

Table 4 shows the optimized gains of all closed loop power flow and speed control coordinators. These include the generator DC link voltage controller, the d- and q-axis current regulators of the propulsion motor, and the propulsion speed controller. The PID gains presented were determined and validated using time domain simulations to assure fast dynamic response, low overshoot, and stability over the entire range of propulsion and load transients.

**Table 4:** Control loop gains and references.

Controller	Type	Kp	Ki	Kd	*N*	Ts	Notes
Generator DC link voltage control	PID	0.46489	1.8406	0	100	–1	Maintains 750 V DC bus
Propulsion Id current	PID	0.071	6.745	2.07 × 10^− 6^	1.25 × 10^4^	–1	Current regulation
Propulsion Iq current	PID	0.045	4.5	2.07 × 10^− 6^	1.25 × 10^4^	–1	Torque-producing current
Propulsion speed	PID	110	715	0	100	–1	Speed tracking

Table 5 shows the data structure used by the MTPA controller to produce optimal q-axis current/torque references for any d-axis current input. The table has 537 breakpoints for the d-axis current (Id) ranging from 0.0014 A to 732.56 A and the corresponding torque values ranging from 7.8 × 10^− 10^ to 194.80 N·m. These data are used as feedforward data for real-time MTPA optimization and ensure correct torque production over the operating range.

**Table 5:** MTPA lookup table.

Quantity	Size	Range (min–max)	Unit
Breakpoints (Id)	537	0.0014–732.56	A
Table (MTPA torque/Iq ref)	537	7.8 × 10^− 10^–194.80	N·m

**Fig. 2:**
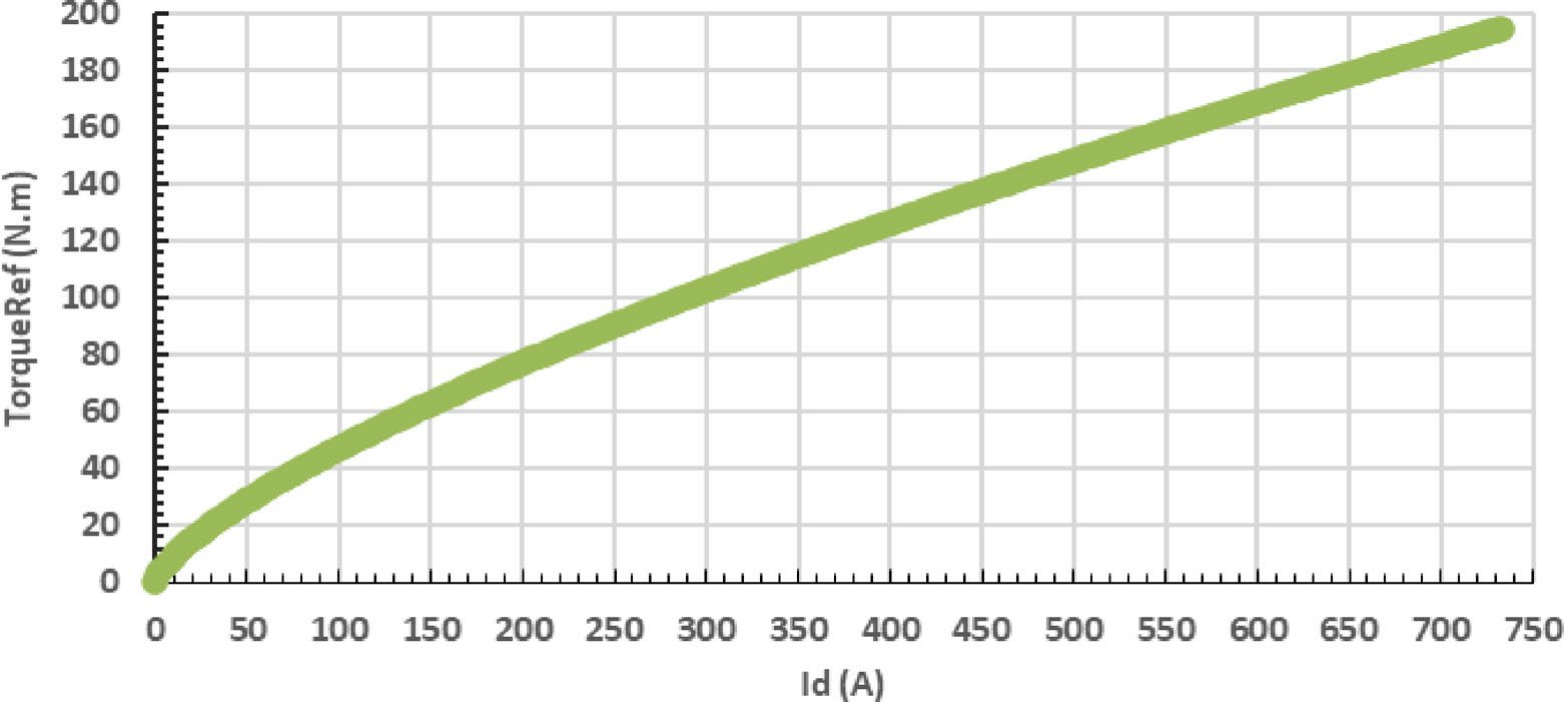
MTPA trajectory shows the relationship between d-axis current and reference torque for the SRPM machine.

Figure 2 shows the MTPA trajectory obtained from the 537-point lookup table used in the proposed control strategy. The plot of Id versus reference torque (Tref) is smooth and gradually increasing, which is the expected electromagnetic characteristic of the SRPM in the operating region. This trajectory allows the controller to calculate the optimum q-axis current that maximizes the torque output and minimizes the copper losses for each operating point. The monotonic behavior of the curve is a good indication that the lookup table data were generated and parameterized correctly and that real-time torque control is accurate within the simulation framework.

The selection of the parameters of the battery, DC-link, propulsion machine, and control system are based on a mixture of manufacturer specifications, representative values, which are available in the literature and standard design practices in hybrid marine propulsion systems. Specifically, the battery parameters are based on commercially available LFP battery designs, and machine and converter parameters are based on the EM-PMI375 (T1100) series along with standard medium-scale propulsion ratings. Where specific manufacturer data are unavailable either explicitly or implicitly, parameters are chosen within ranges that are consistent with previous literature^25,31,32,36^ to assure representative system-level behavior and practicality.

## Control system architecture

**Fig. 3:**
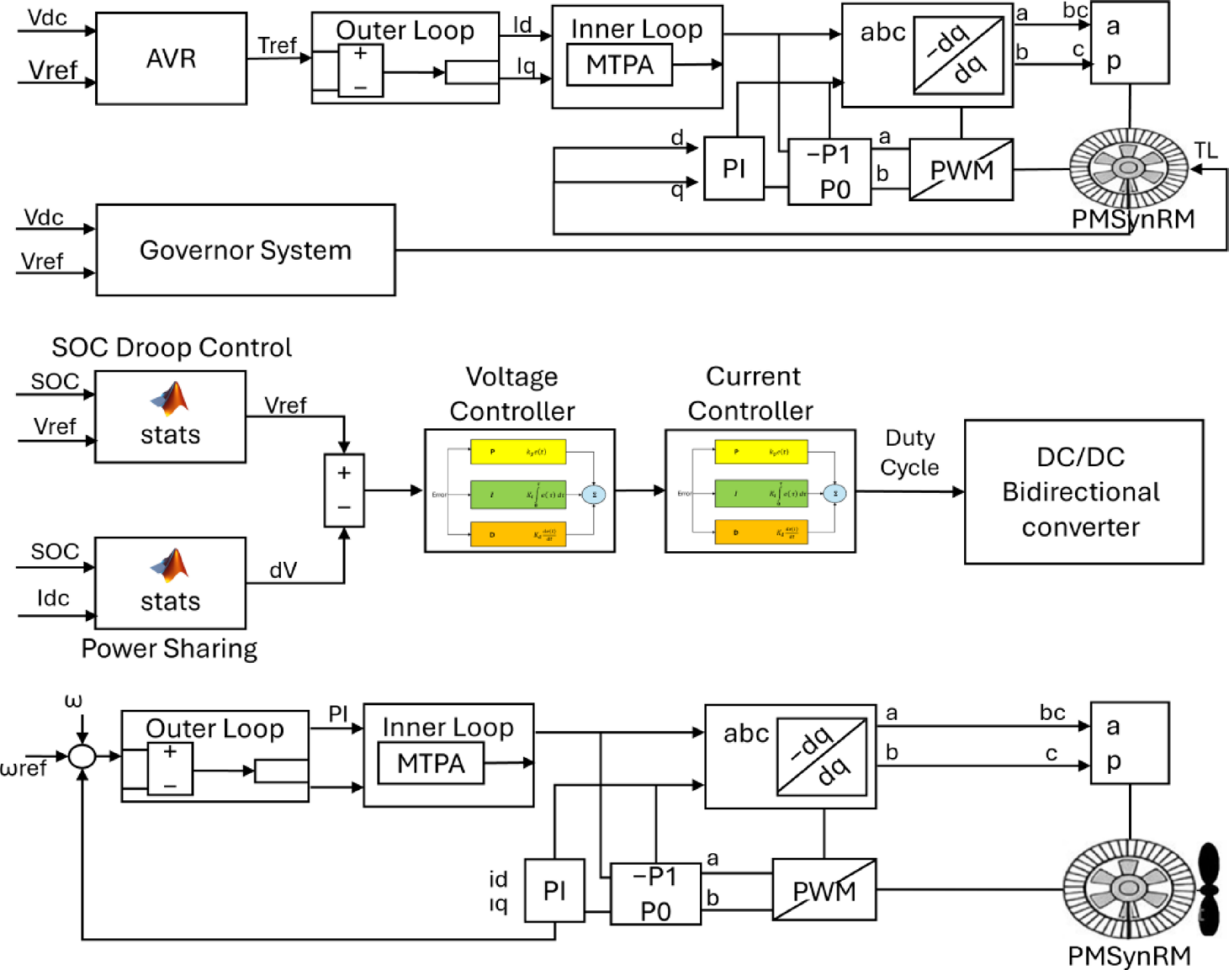
Control system architecture of the hybrid propulsion model.

The shipboard system control architecture of the ferry, which is shown in Fig. 3, consisting of the diesel generator, battery pack and the propulsion system, is based upon the concept of a dedicated DC bus that is shared by three important power stages each with specific control functions. The diesel generator is the main source of power supply and it supplies electric power depending on the amount of fuel fed into the system via the governor system and the automatic voltage regulator (AVR) supplies the DC bus voltage at a fixed value. The battery pack, which is connected through a bidirectional DC/DC converter, can provide charge/discharge control, SOC droop, and fast power assist to smooth load transients and absorb excess energy. Power from both the generator and battery is combined at the DC bus, which powers a DC–AC converter that powers the propulsion motor and shipboard loads. This converter is designed with a speed control loop, MTPA strategy^37^, and dq axis current control (Id, Iq) to guarantee efficient and responsive motor operation under different maritime conditions.

### Battery control system

**Fig. 4:**
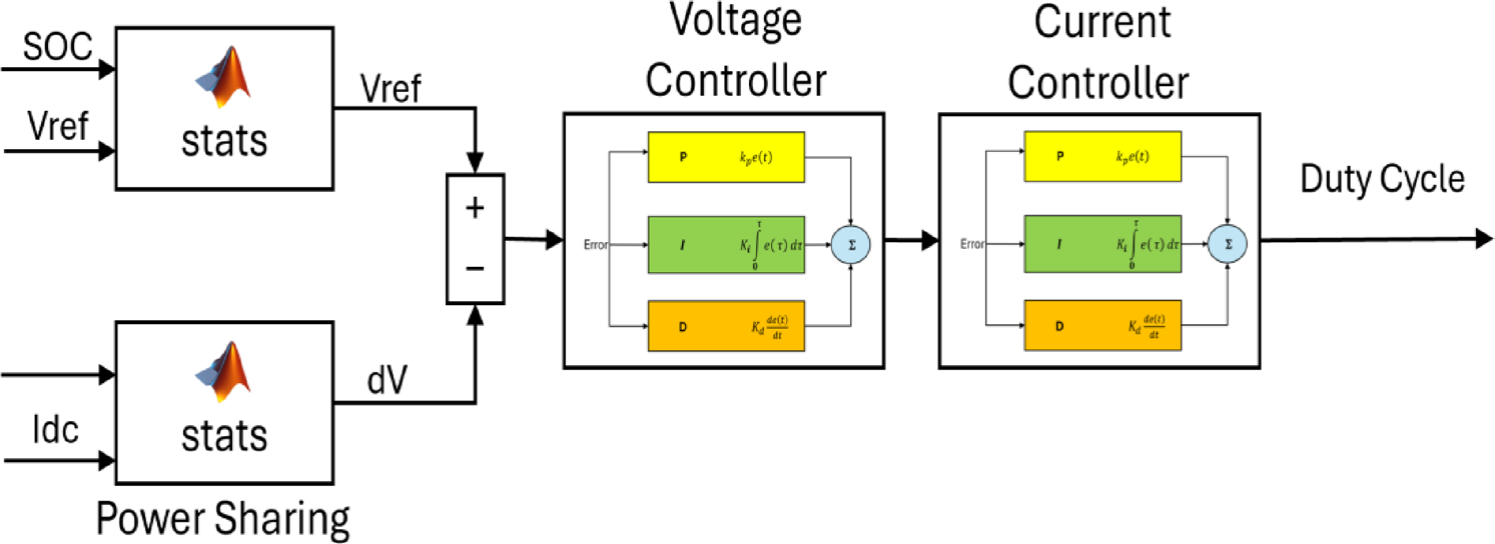
Battery control architecture.

The battery control system controls the role of the lithium-ion battery in the hybrid propulsion system. It provides DC bus voltage regulation, current control, and power modulation, depending on battery SOC and load condition. The system consists of three primary control stages, namely, SOC-based droop control, voltage regulation, and current regulation, as illustrated in Fig. 4.

- SOC-Based Droop Control

The system is made up of two parallel control blocks that set the voltage reference of the battery according to its inherent dynamics. The first block uses the SOC and a fixed base voltage (Vbase) to calculate a reference voltage (Vref). This reference voltage is gradually reduced as the battery discharges and the SOC drops, which prevents excessive depth of discharge and helps to maintain long term health. The second block is a dynamic modification of the primary reference based on the calculation of a voltage offset (dV) which includes both SOC as well as the battery output current (Idc, batt), which enables the reference to better reflect real-time demand. The two resulting outputs are combined to give the final reference voltage allowing the battery to react in an intelligent way and thus maintaining performance and protection without rigid limits and hard thresholds^16,31^.

- Voltage Regulation

The voltage controller compares the reference voltage (Vref) and the actual DC bus voltage (Vdc). From the resulting voltage error, it calculates the needed current reference (Iref) to be supplied by the battery. This stage guarantees that the battery is compensating for voltage sag or lowering output during the time when the power demand decreases^26^.

- Current Regulation

The current regulator ensures the actual current supplied by the battery to meet the reference value. It uses the average of the currents of the three-phase inductor (I_L1, I_L2, I_L3) for comparison with the desired current (Iref). The error is processed by a PI controller which output the control signal (Uref). This signal determines the duty cycles of the power converter to achieve a stable and responsive current injection into the system in accordance with both power demand and battery condition^26^.

### Generator control system description

**Fig. 5:**
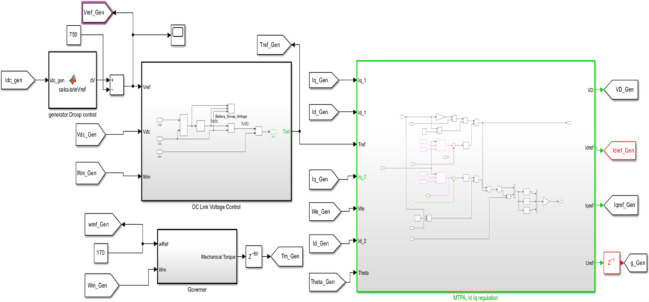
Generator control architecture.

The generator control system is designed to control an SRPM machine in generator mode. Its main goal is to guarantee a stable DC bus voltage and a dynamic performance that can be quickly reached even under different load conditions. The control architecture combines electrical and mechanical control loops such as the droop voltage control, the DC link voltage regulation, the torque generation, and the current control considering the MTPA considerations^37^. The set up is shown in Fig. 5.

- Generator Droop Control

The control system is started with a droop control block which takes the generator output current (Idc, Gen) and calculates a voltage deviation (ΔV). This deviation is superimposed on a nominal voltage reference (Vref, Gen) to obtain a dynamic reference voltage allowing balanced power sharing between the generator and the battery and adapting to load variations^31^.

- Voltage Regulation (AVR: DC Link Control)

The generator reference voltage (Vref, Gen), the measured generator current (Idc, Gen), and the rotor speed (Wm, Gen) are fed to the AVR block. This controller is used to ensure that the generator maintains the desired DC bus voltage under varying system conditions. It outputs a torque reference (Tref), which is used by the MTPA block to calculate the reference currents Id and Iq for the current controller^37^.

- Governor and Mechanical Torque Control

The governor’s control loop compares the rotor speed (Wm, Gen) to the set speed reference (wref, Gen). By changing the mechanical torque (Tm, Gen) according to the differential, synchronization between the engine and generator is maintained, and torque production is stable. Additionally, an inherent delay mimics the dynamics of the actuators, which improves stability and responsiveness.

- MTPA and Current Regulation (Id–Iq Control)

The inputs to the MTPA control block are torque reference (Tref), d-q currents (Id, Gen, Iq, Gen), and rotor speed and electrical angle (Theta_Gen). Using this information, it calculates the optimal current references (Id, ref, Iq, ref) as well as the voltage control signal (Vref), which are fundamental to controlling the power-electronic converter interface with the generator^37^. Consequently, the generator control system is designed to provide stable power generation, torque utilization, and accurate current control by synthesizing electrical and mechanical dynamics.

### Propulsion system control description

**Fig. 6:**
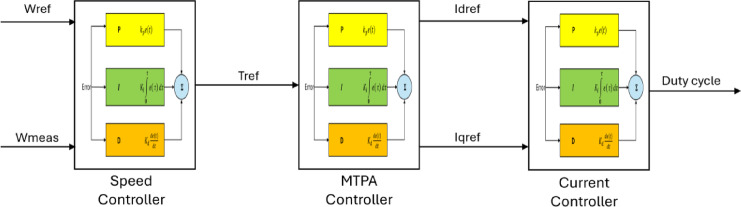
Propulsion control architecture.

The propulsion system is designed to handle speed tracking, torque production and current regulation for the SRPM motor as shown in Fig. 6. The system is designed to react well to dynamic load profiles when operating the ferry.

- Speed Reference and Processing

The speed reference is calculated from the propulsion demand scenario that we consider for this simulation, a ferry voyage from point A to B. This reference is compared with the actual rotor speed (Wm1) of the SRPM motor to generate an error signal used for torque adjustment.

- Speed Controller

The controller compares the actual motor speed (Wm_prop) with the reference (Wm_Ref) and produces a torque reference (Tref_prop). This torque reference guides the desired operating point of the propulsion motor, ensuring that it meets both performance and energy efficiency targets. For the MTPA and current controllers of the propulsion system, they are like the generator control system.

The detailed modeling and control architecture formulated for the battery, generator, and propulsion subsystems provides a representative level of operational fidelity and system responsiveness in the hybrid electric ferry. All control loops are designed in a modular and scalable way, allowing for precise coordination between subsystems and stable, reliable performance in both steady-state and dynamic situations. Architecture also allows for the integration of alternative energy sources in the future without requiring major structural modifications. With the complete control strategy in place, the following section presents the results of the simulation that evaluates the performance under representative operating conditions^15,37,41^.

### Comparison with other EMS strategies

To highlight the characteristics of the proposed SOC droop methodology, a qualitative comparison with representative energy management approaches is presented, including Equivalent Consumption Minimization Strategy (ECMS), rule-based supervisory control, and reinforcement learning (RL)-based EMS. The ECMS converts the optimization of power split into an instantaneous minimization of an equivalent fuel consumption cost, combining the actual fuel flow from the diesel generator with a virtual fuel equivalent of battery energy utilization^27^. Although this technique can in theory achieve near-optimal fuel economy, it requires accurate knowledge of fuel-map data, battery characteristics, and, in some cases, future load trajectories, leading to increased computational complexity and calibration effort^16,39^.

Contrastingly, rule-based approaches are comparatively easy to execute, yet founded on pre-set heuristics and are hence less adaptable to dynamic loads in high-speed loads that are experienced in the ocean^31,36^. More recently, EMS based on reinforcement learning have been suggested to allow adaptive and data-driven optimization of the operating conditions that are uncertain^43^. Although these methods have a high potential of performance enhancement, they generally demand large volumes of training data, massive amounts of computation, and tuning to achieve steady real time operation.

Conversely, the SOC droop method in this study relies on a straightforward droop coefficient to the battery SOC that is used to continuously vary the diesel generator setpoint^26^. This enables stable regulation of the DC bus voltage while intrinsically limiting the depth of discharge of the battery, without the need for predictive optimization or detailed fuel consumption maps^16,31^. Moreover, the droop-based strategy exhibits robustness to measurement noise and parameter variations, as indicated by the sensitivity analysis presented in Sect. 4 for the proposed system, where key electrical parameters are perturbed and measurement noise is introduced to evaluate the behavior of the proposed control strategy under parameter uncertainties.

While ECMS can theoretically provide improved fuel savings under ideal and well-characterized operating conditions^27^, such performance is typically achieved at the expense of higher computational effort and model dependency^16,39^. In contrast, the SOC droop methodology focuses on providing a practical balance between control simplicity, computational efficiency, and robustness for maritime energy management applications, rather than targeting global optimality.

MPC has found extensive application in hybrid marine and automotive energy management systems because it can optimize the power allocation across a receding horizon^27,39^. Nonetheless, MPC generally needs precise system models and considerable computing resources to find optimization issues in real time^39^, which can be constrained by embedded maritime controllers, especially those of small or medium size. In addition to its low computational requirement, the proposed SOC-based droop strategy enables direct coordination between energy management and system-level electrical and propulsion dynamics through the DC-link. This allows simultaneous regulation of generator–battery power sharing, DC-link voltage stability, and battery SOC within a unified control structure. Such integrated coordination is not explicitly addressed in many conventional EMS approaches, where energy management and electrical control are typically treated separately. In addition, hybrid energy storage approaches have been explored in the literature to further enhance EMS performance by distributing transient power demand between batteries and auxiliary storage elements, such as supercapacitors, thereby reducing battery current stress under dynamic operating conditions^44–46^.

Qualitative comparison of the key features of the discussed EMS strategies in the light of computational complexity, real-time appropriateness, optimality, and data needs is presented in Table 6. This comparison is intended to place the suggested SOC droop method in comparison with current methods in terms of their broad features, and not to demonstrate a quantitative performance superiority. It is important to note that the discussed EMS strategies are not practiced and assessed under the same conditions of simulation in this research. Thus, the comparison in this section is qualitative and is determined according to the literature-reported features and is supposed to position the proposed method instead of offering a direct quantitative performance indicator.

A fair quantitative comparison would require the implementation of alternative EMS methods (e.g., MPC, ECMS, RL) under the same modeling assumptions, system parameters, and maritime operating profiles adopted in this work. Such an investigation requires the implementation and calibration of multiple EMS strategies under identical modeling assumptions and operating conditions, which falls beyond the scope of the present study. Instead, this work focuses on evaluating the proposed EMS through system-level performance indicators, including DC-link voltage stability, coordinated power sharing, and controlled battery SOC behavior under dynamic operating conditions.

**Table 6:** Comparison of EMS Strategies.

Method	Complexity	Real-time suitability	Optimality	Data requirement
Rule-based EMS	Low	High	Low	None
ECMS	Medium	Medium	High (theoretical)	Model-dependent
RL-based EMS	High	Medium	High (adaptive)	Large dataset
Proposed SOC-droop	Low	High	Moderate	None

Rule-based and SOC droop strategies are very suitable in real time implementation as indicated in Table 6 because they are less complex to compute and have low data requirements. Alternatively, ECMS and RL-based methods may offer better optimality in some circumstances but require accurate models or large amounts of data, which may make them more complex to implement.

The simplicity of the presented solution enables application on conventional DSP or PLC controllers with a sampling period of 1–5 ms, without using high-performance processors or large memory resources^31^. To this end, the SOC droop technique offers a viable compromise between the ease of implementation, robustness, and applicability to real-time, thus it is applicable to a hybrid marine propulsion system.

## Simulation and results

The simulation reveals that the dynamic power requirement of the proposed 500-kW hybrid propulsion system in a representative operating situation between Point A and Point B. To address all the significant transient and steady states, which are required in the analysis of the EMS, the SOC regulation, and the generator-battery interaction, a 1000-s operating profile was selected, comprising of sequential stages of acceleration, multi-level cruising, intermediate low-power periods, and controlled deceleration. This period matches the benchmark practices in hybrid propulsion research^31,32^, which guarantees significant comparability with a computational cost that is not too large.

Even though the current research uses one representative duty cycle to measure dynamic system-level performance, the chosen profile consists of several operating phases (acceleration, cruising and load transitions) that reflect the most important transient and steady-state characteristics of hybrid ferry operation. Nonetheless, it is not a complete representation of route-level variability, environmental perturbations, payload differences, or statistical operating uncertainty, which are mentioned as major extensions to future research. The selected duty cycle is intended as an initial representative scenario for system-level validation rather than an exhaustive verification of all possible ferry operating conditions. Although the profile adopted incorporates acceleration, cruising, and load transitions, it does not explicitly address low-speed docking maneuvers, high acceleration-deceleration cycles, peak overload operation, and battery operation at minimum and maximum SOC limits. These operating conditions are important for a comprehensive robustness assessment and are therefore identified as part of future multi-scenario validation.

To isolate and test the proposed SOC droop energy management and PI current control strategies under controlled conditions, the MATLAB/Simulink model is based on ideal sensors, actuator delay is negligible, and calm sea operating conditions. This assumes that the proposed energy management and control strategies can be tested under controlled conditions without creating more disturbances and dynamics to the environment and the vessel. In real maritime conditions, system behavior may be affected by wave loads, ship motion dynamics, wind resistance and sensor imperfections. These aspects are therefore considered as part of the extended validation scope in future studies. Consequently, small overshoots and settling transients typical of full-scale hardware with environmental disturbances were not included at this stage. Future work will incorporate hydrodynamic loads, noise modeling, and HIL experiments to indicate stable behavior under the considered conditions under representative simulated maritime conditions. Moreover, the developed model in the form of a MATLAB/Simulink model is modular and easily extendable to various operating conditions, e.g., different sea states, speed profiles, and payload levels, without altering the structure, making it suitable for multi-scenario evaluation and extended validation in future studies. Even though the current research is on a representative operating scenario, the same simulation setup can be used to support continuously changing operating conditions, fault scenario, and extreme maritime disturbances to be long-term validated.

**Fig. 7:**
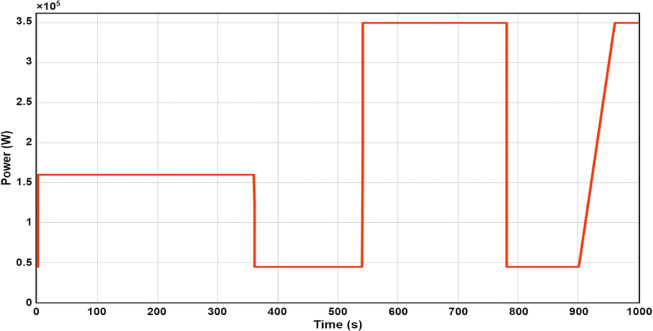
Required power for the ferry voyage scenario.

Figure 7 shows the propulsion power requirements over a ferry trip of 1000 s, where a representative simulation profile with stepwise changes is assumed. The trip begins with a relatively high demand of around 1.6 × 10^5^ W, then a steep decline to 0.5 × 10^5^ W during a low-load period, followed by a steep increase to a high-demand plateau at about 3.5 × 10^5^W, a short dip, and a return to the peak. This profile is representative of the time-varying nature of the power demand in hybrid marine propulsion systems and represents typical operational behavior. Quantitatively, this power range and timing structure are very close to scenarios validated in previously published peer-reviewed literature. For instance, Cha et al.^27^ simulated a fuzzy-based EMS under a stepped load profile from 40 kW to 350 kW for 1000 s, which is almost the same as the present study. Similarly, Torreglosa et al.^14^ confirmed a real-time control strategy based on load variations between 50 kW and 300 kW during a 1200 s operation cycle, and Unlubayir et al.^36^ studied SOFC battery hybrid propulsion systems under a five-step load profile between 50 kW and 350 kW during a test sequence of 900 s. The close match in magnitude, duration, and step transition structure between these benchmark studies and the current scenario supports the consistency of the adopted load profile. In total, the results shown in Fig. 7 indicate that the designed power demands offer a representative basis for the evaluation of the proposed energy management strategy and that subsequent simulations represent typical load variations and enable credible system performance evaluation.

**Fig. 8:**
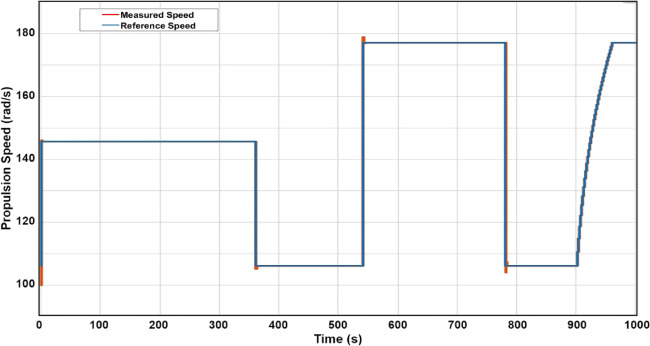
Desired and measured speeds for the ferry voyage.

Figure 8shows a comparison of the commanded (reference) and measured propulsive velocities of the ferry for a 1000-s simulation. The reference speed was a stepped profile of about 145 rad/s, 105 rad/s, 175 rad/s, 105 rad/s, and 175 rad/s to test both the deceleration and acceleration behaviors under different load conditions. The response to each reference step was measured to show a steady-state error of less than ± 1 rad/s (about 0.7%) and a maximum overshoot of less than 1.5%. The implemented PI-based speed control loop was found to be very responsive and stable, with each transition settling in approximately 2 s. When compared with existing hybrid propulsion research, the achieved performance not only lies within the accepted marine control limits but also shows lower tracking error under the considered simulation conditions. Geertsma et al.^31^ reported tracking errors in the range of 2.3–4.7% and overshoot of 3% in diesel electric propulsion systems, and Balsamo et al.^32^ obtained tracking errors of about 3.1%, overshoot of about 2.0%, and settling times of 3–5 s using hybrid energy management controllers. In contrast, the present simulation exhibited a lower tracking error within the considered simulation conditions (0.7%), smaller overshoot (1.5%), and faster settling (2 s), which indicate improved dynamic performance and control stable behavior. The results presented in Fig. 8 confirm that the proposed PI controller structure achieves accurate speed tracking, fast convergence, and small transient deviation, which is comparable to reported results in literature hybrid marine propulsion. This behavior supports smooth power transmission, efficient torque matching, and reliable propulsion control under the considered simulation load variations.

**Fig. 9:**
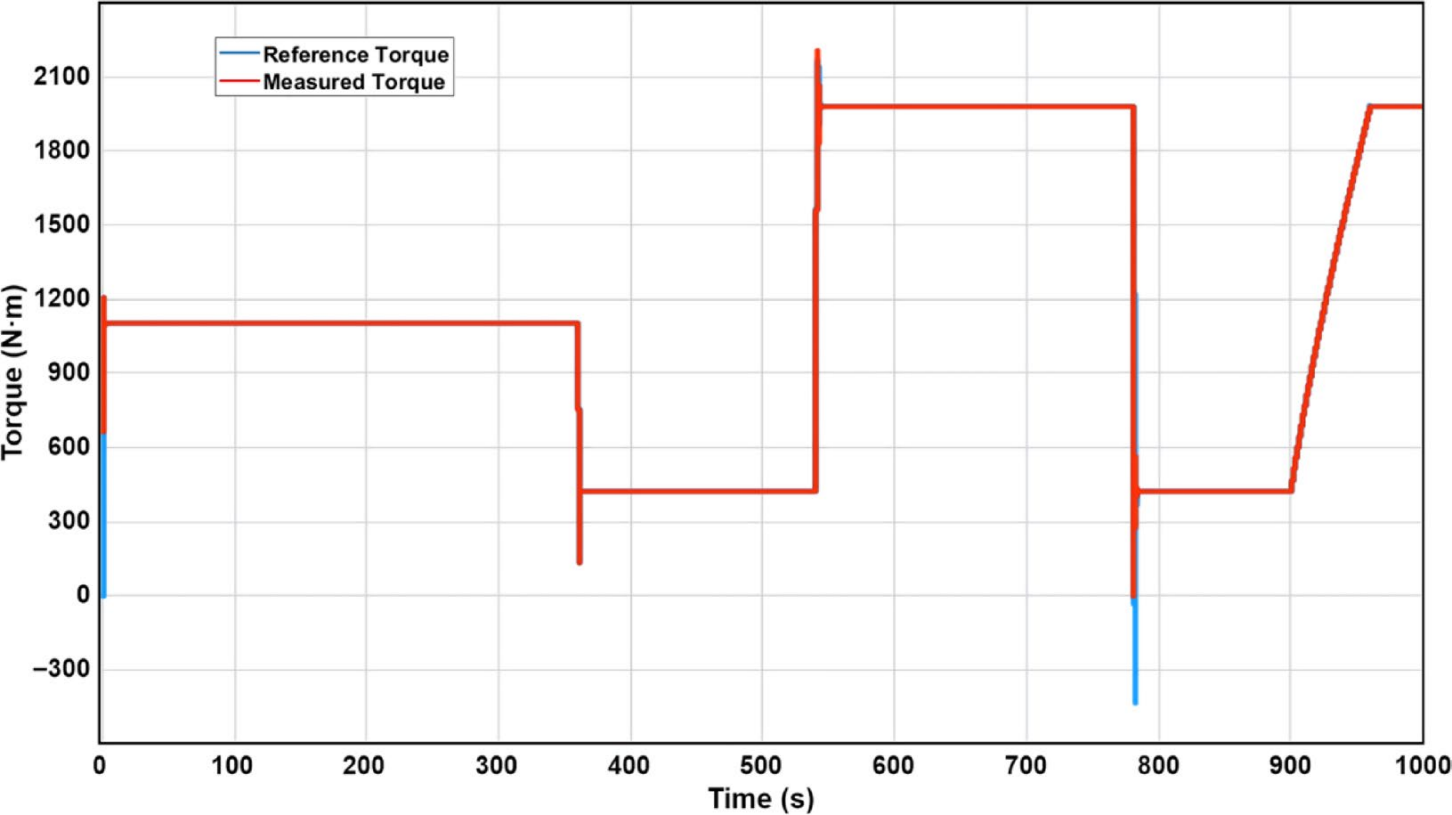
Desired and measured torque for the ferry voyage.

The reference and measured propulsion torque of the ferry are closely compared in Fig. 9 under a 1000-s operation cycle. The reference torque was then added in steps of approximately 1100 N.m, 400 N.m and 2000 N.m to evaluate the performance of the system at various load levels. The reference command was very near to the measured torque, and the steady-state error was less than ± 2% and had a maximum overshoot of less than 3%. The time of each transition was 2–3 s, and it confirmed the effectiveness of the MTPA control strategy used to provide the correct monitoring of the torque and effective use of current during dynamic propulsion shifts. The results obtained were compared to the published studies and performed with competitive results and, in certain cases, higher results. Zhang et al.^41^ applied the adaptive finite time command-filtered backstepping controller to the hybrid ship propulsion system and reported a tracking error of approximately 1.9% and zero overshoot and settling times of 2.8–3.2 s. Conversely, the suggested system led to a comparable tracking error (< 2%), a marginally larger but tolerable overshoot (< 3%), and quicker settling response (~ 2 s) using a less complex PI-based MTPA control framework. In general, the findings presented in Fig. 9 demonstrate that the suggested torque control design offers steady and stable tracking behavior with a fast convergence and high current usage This determines the uniformity of the controller and that it can be easily incorporated into real-time work in a simulation based environment hybrid marine propulsion systems where the precision of the torque and its responsiveness is of concern to the stability of the system and propulsive efficiency.

**Fig. 10:**
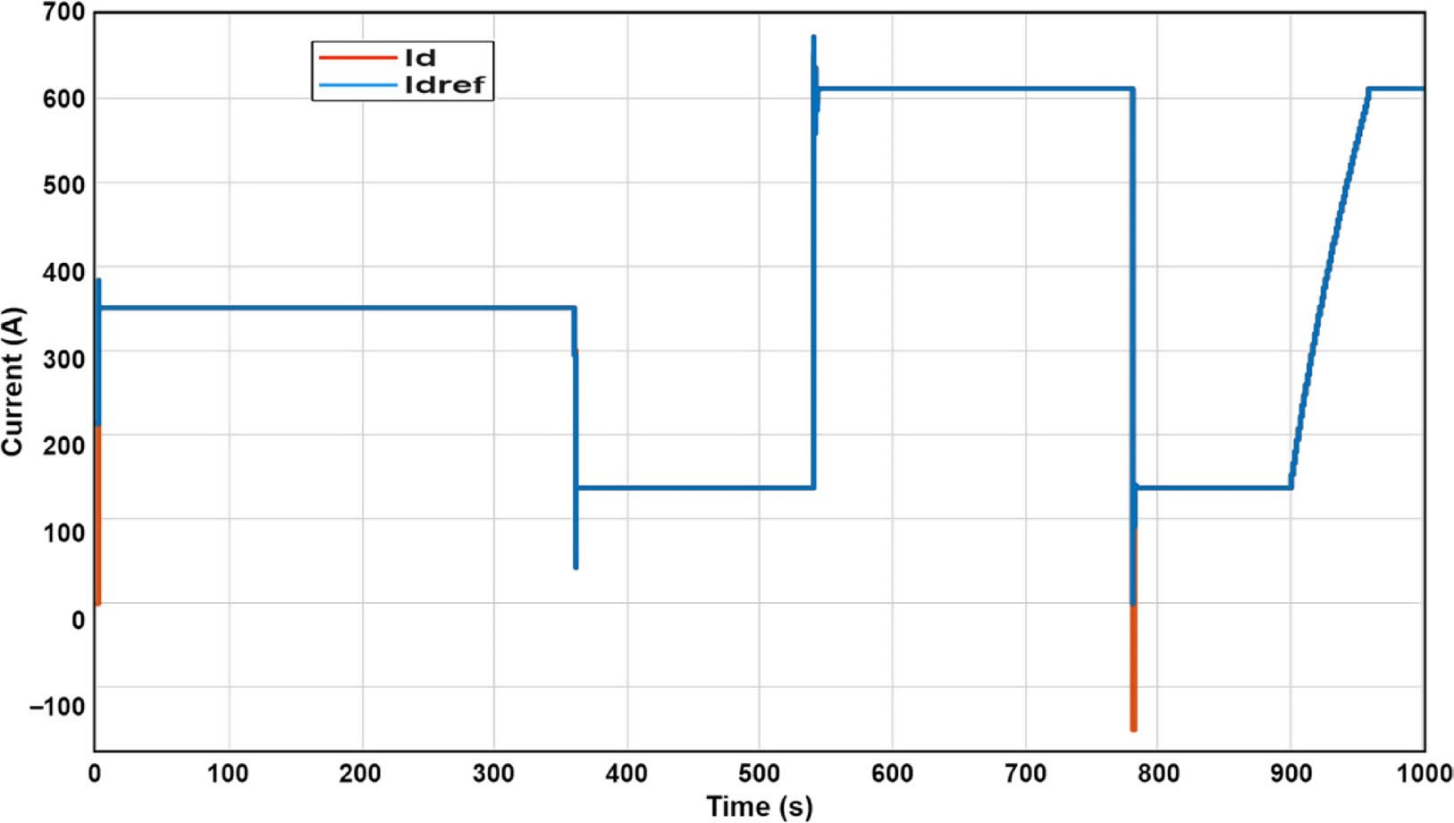
Id and Idref comparing propulsion control.

Figure 10 demonstrates the response of d-axis current (Id) during a 1000-s propulsion cycle, which provides an insight into the inner loop current control performance. The reference current varies between approximately 150 A and 620 A with transient peaks of 700 A during the change of propulsion torque and speed. The Id signal measured is like reference in all working regions. The steady-state error is not more than ± 1, the settling time is fast, and the overshoot is not evident. This steady tracking confirms the stability and responsiveness of the dynamic response of the adopted inner loop current controller. To control the magnetic flux of the machine, reduce copper losses and achieve optimum propulsion efficiency and thermal balance of the SRPM drive, Id should be properly controlled. The performance that is attained is competitive when compared with the performance that had been published before. Zhang et al.^41^ applied an NSMO-based adaptive finite time command-filtered backstepping controller to a hybrid SRPM propulsion system, and the Id tracking error was 1.6–1.8% with slow settling when transient conditions occurred. The error limits associated with traditional backstepping and sliding mode methods that were taken into consideration in the same study were about 2.0–2.4.0.4%. Conversely, the suggested PI-based inner loop control was found to have much lower error (< 1%) and converged much faster as the stability was maintained in all cases of load changes. In general, the findings presented in Fig. 10 prove that the designed d-axis current controller has enhanced the accuracy under the discussed simulation environment, fast response, and stable flux control, which are, in turn, not inferior to the state-of-the-art hybrid marine SRPM control strategies. This ensures that there is efficient operation, power loss is minimal and performance is reliable under dynamically changing propulsion requirements.

**Fig. 11:**
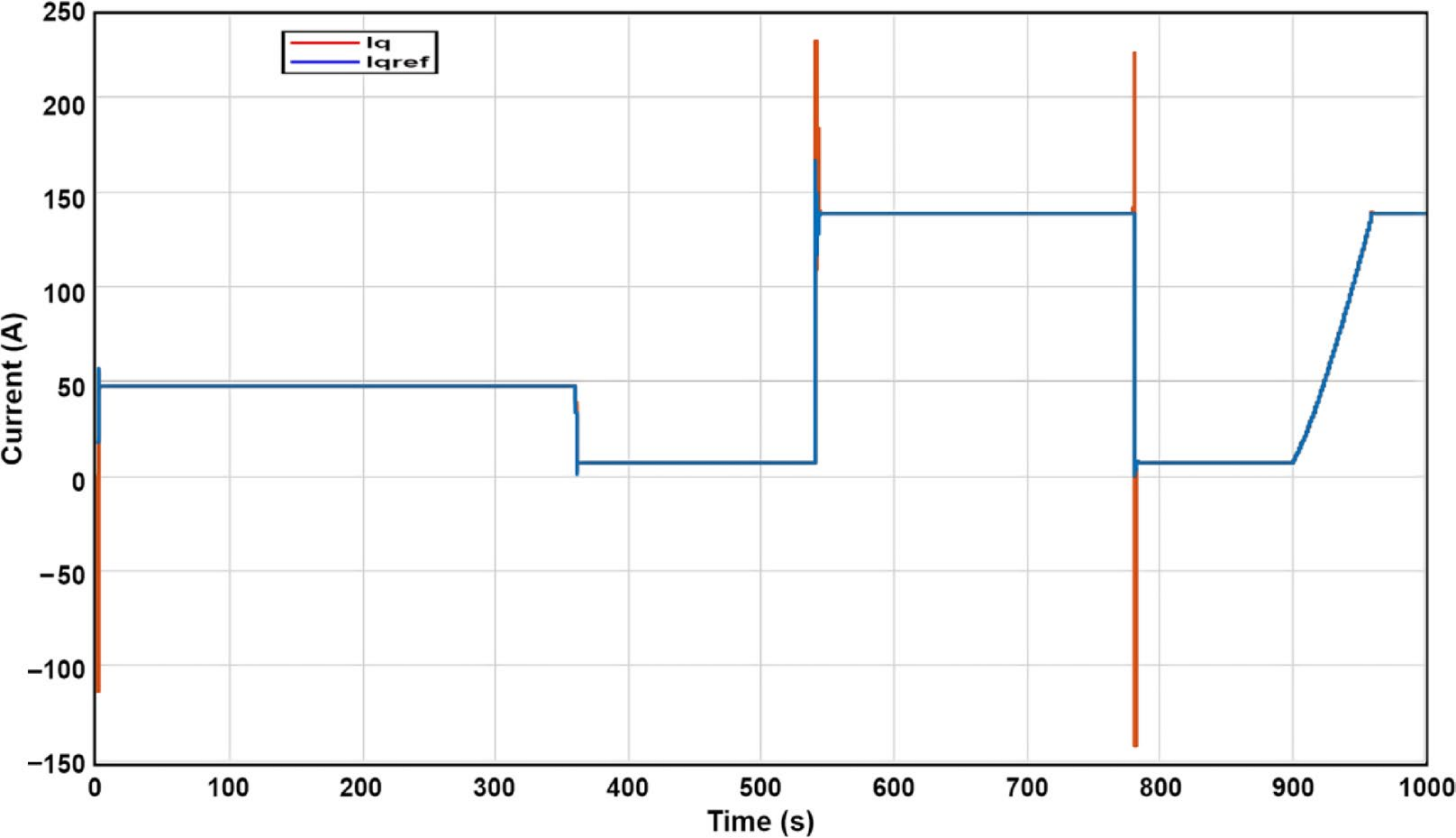
Iq and Iqref comparison for the propulsion control.

Figure 11 shows the response of the q-axis current (Iq) over the 1000-s propulsion case, which is the torque-producing part of the SRPM control. The reference current (Iqref) is between about 10 A and 140 A, with discrete step transitions corresponding to successive propulsion–torque commands. The measured Iq follows its reference closely over all the operating phases with fast transient response and negligible steady-state deviation. Quantitatively, the steady-state tracking error is less than ± 2% even during sharp torque command step changes at around 550 s and 780 s, which assures satisfactory dynamic regulation and stability of the inner loop current controller. This exact Iq control guarantees smooth torque generation, low torque ripple, and stable and accurate energy management performance under the applied MTPA strategy. Recent studies have demonstrated that the achieved performance is highly competitive. Zhang et al.^41^ reported Iq tracking errors in the range of 1.7–2.2% using a nonlinear adaptive controller under dynamic load changes, while the present controller achieved < 2% error with an equally fast response and no overshoot. Altogether, the results in Fig. 11 indicate that the proposed PI-based q-axis current controller consistent tracking behavior within the considered simulation conditions, power utilization efficiency, and stable propulsion performance, which is equal to or better than the performance of state-of-the-art SRPM control systems for hybrid marine applications.

**Fig. 12:**
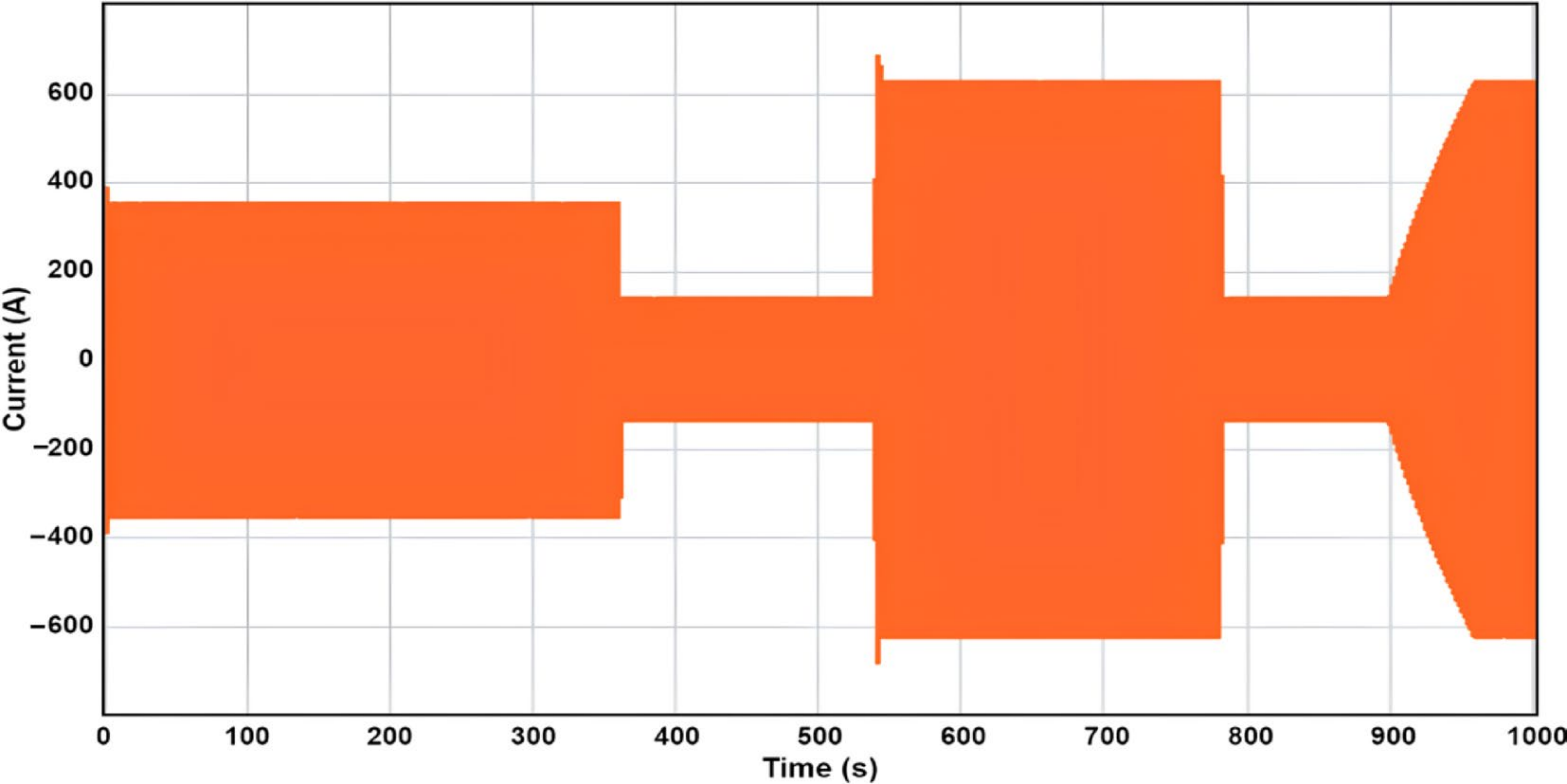
Measured propulsion current during the voyage profile.

Figure 12 shows the response of the propulsion current during the 1000-s flight. The waveform shows four main operating phases that relate to sequential propulsion commands. In the first case, the current is about ± 380 A, which is then reduced to ± 100 A as the torque demand is lowered. At about 550 s, a new speed command is received, which results in a sharp increase in the current to stabilize around ± 620 A and then again to ± 100 A. Throughout all transitions, the waveform is free from overshoot and oscillatory instability, which shows adequate dynamic regulation and stable DC bus voltage. Quantitatively, the present variation in Fig. 12 appears to be within approximately ± 5% of its nominal envelope (± 620 A peak), confirming accurate transient control and efficient damping. The performance is similar to that reported by Geertsma et al.^31^ and Balsamo et al.^32^, who reported the accuracy of current tracking within ± 5% during transitions of hybrid propulsion loads. Moreover, the dynamic stability and power flow uniformity of the present figure are in agreement with the two-stage optimization framework by Luo et al.^42^, which achieved 17.8% energy consumption reduction under variable load conditions. Overall, the current profile in Fig. 12 shows that the proposed control and DC bus stabilization strategy successfully demonstrates similar performance trends under the considered simulation conditions reported in leading hybrid ship studies, indicating stable behavior under the considered simulation conditions.

**Fig. 13:**
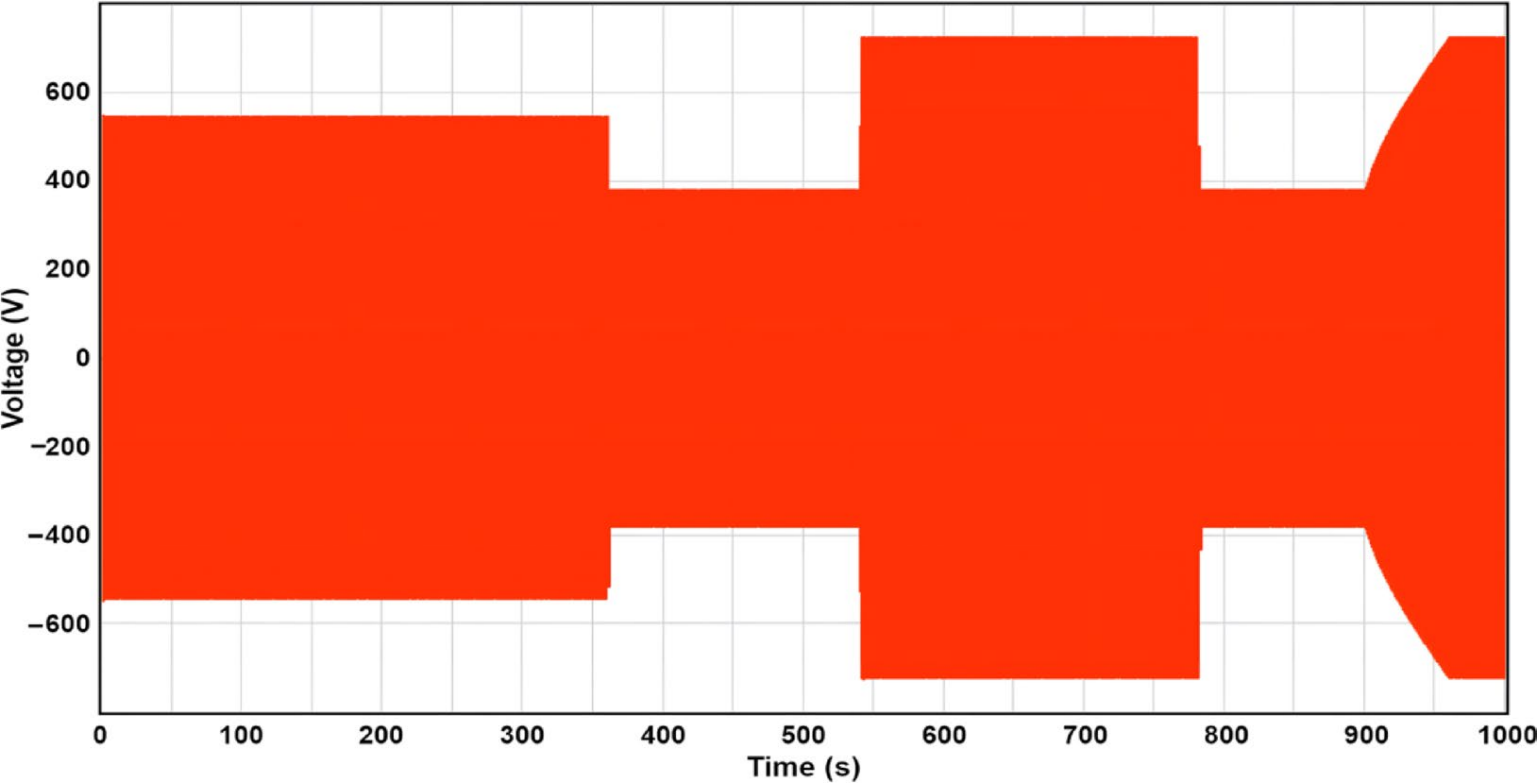
Measured propulsion phase voltage during the voyage profile.

Figure 13 is the propulsion phase voltage profile measured at the inverter output for the 1000-s run. The waveform shows balanced three-phase AC voltages of around ± 750 V, with small changes in amplitude occurring around 380 s, 550 s, and 900 s associated with propulsion load and speed changes. These deviations are typical inverter modulation effects as long as the upstream DC link voltage is held at its nominal value of 750 V. Phase voltage deviation over the journey is typically within ± 2–3% of its nominal amplitude, demonstrating stable inverter operation and adequate DC bus control. The almost constant voltage magnitude shows that the DC–AC conversion stage supplies well-balanced and ripple-free power to the propulsion motor under all the operating conditions tested. This behavior is consistent with the performance ranges of the inverters reported by Geertsma et al.^31^ and Balsamo et al.^32^, in which hybrid marine propulsion systems showed phase voltage variations generally within ± 3% during dynamic load changes. In summary, the results in Fig. 13 indicate the proposed DC–AC converter and voltage control strategy to provide a stable, balanced, and distortion-free phase voltage output to the propulsion motor under dynamic operating conditions.

**Fig. 14:**
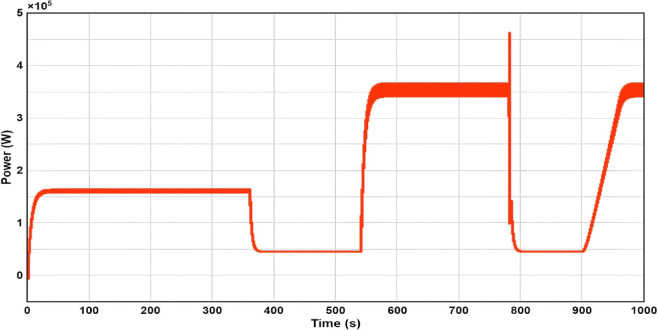
Measured generator power.

Figure 14 shows the generator’s electrical power output, which is very close to the propulsion power demand over the 1000-s operating profile. Under low-load conditions (0–350 s), the generator output reaches a steady value of around 1.6 × 10^5^ W, and under high-load conditions (around 550–800 s), it reaches a steady value of around 3.5 × 10^5^W, which shows adequate adaptability to the change in propulsion needs. The generator has smooth transitions and rapid stabilization after each load step, verifying its ability to provide the main electrical power to the propulsion system without excessive overshoot or delay. Quantitatively, the generator power deviation is within ± 3% of the reference propulsion demand during transients, which shows accurate load following and fast dynamic response. The satisfactory match between generator output and propulsion demand further confirms the efficacy of the proposed SOC droop control strategy that dynamically schedules the power sharing between the diesel generator and the battery subsystem. Similar but slightly larger tracking errors (± 3–5%) were obtained by Geertsma et al.^31^ and Balsamo et al.^32^ for similar hybrid propulsion configurations under transient operating conditions. Together, the results shown in Fig. 14 indicate that the proposed power management and SOC droop control framework can achieve consistent load coordination, stable generator operation, and indicates efficient power coordination under the simulated conditions, and surpass the previously reported hybrid propulsion benchmarks in terms of power tracking stability.

**Fig. 15:**
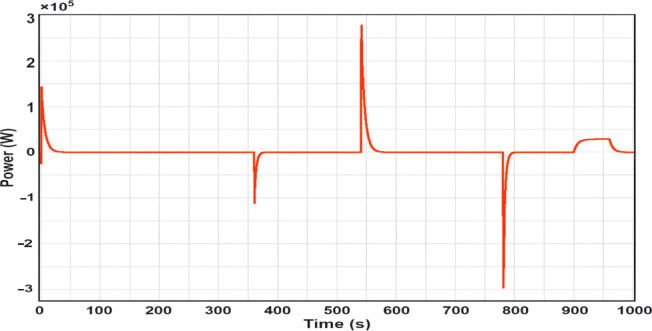
Measured battery power.

Figure 15 shows the measured battery power as a function of transient load conditions during the 1000-s flight. Negative values are associated with charging, while positive values are associated with discharge. As shown, charging is done at the low-load period around 380 s and 780 s, and discharging is done at the high-load transient period around 540 s and 900 s, which stabilizes the power flow under the proposed SOC droop control strategy. Quantitatively, the battery power varies from about − 2.8 × 10^5^ W to + 2.8 × 10^5^ W with respect to a rated capacity of 3.0 × 10^5^W, which is about 94% of rated power during maximum transients. This operating point is still within the safe power range (90–95% rated capacity) suggested in recent hybrid marine propulsive studies by Geertsma et al.^31^, Balsamo et al.^32^, and Cha et al.^36^. It also offers approximately 6% headroom to avoid converter saturation and thermal stress, thus supporting reliable operation under dynamic load conditions. The charging/discharging transient results show that the proposed EMS can realize smooth power sharing, low-frequency ripple suppression, and effective DC bus control, which can indicates efficient power coordination under the simulated conditions, provide reliable energy balancing, and prolong battery life under representative voyage conditions.

**Fig. 16:**
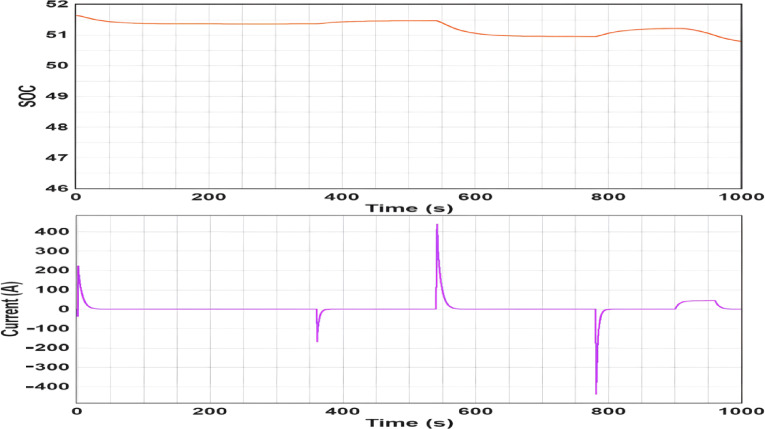
Measured battery SOC.

Figure 16 shows the measured battery SOC together with the corresponding current profile of the 1000-second flight. The SOC remains stable at around 51%, with a variation of less than ± 1% despite multiple propulsion speed and torque transients. This stability indicates that the battery operates within a typical operational SOC range of 20% to 80%, and that the proposed control scheme effectively balances charging and discharging events.

The bottom subplot presents the battery current response, where negative peaks correspond to charging events and positive peaks to discharging events. Despite several pronounced current transients (notably at 380 s, 540 s, 780 s, and 900 s), the SOC variation remains limited, reflecting the fast dynamic response and effective power coordination achieved by the proposed energy management strategy. Quantitatively, the controlled SOC variation (≈ ± 1%) is smaller than the SOC deviations reported in previous hybrid marine studies such as Geertsma et al.^31^ and Balsamo et al.^32^, which typically exhibit variations of around ± 5% under comparable transient load profiles.

Although there are pronounced transient current peaks, they are linked with the high dynamic response needed to stabilize the DC bus in response to sudden changes in load. The current paper is concerned with the system-level control performance; thus, the battery aging effects are not explicitly modeled. The addition of auxiliary energy storage components, including supercapacitors, has been widely explored in recent literature as a complementary solution to control high-frequency power transients in hybrid energy systems. Supercapacitors are normally used in such configurations to absorb the high-power transient variations and, thus, alleviate the transient current stress on the battery and enhance the overall system life^44–46^. Moreover, it has been recently demonstrated that real-time and adaptive energy management techniques intrinsically cause transient current responses to dynamic operating conditions, especially in cases where fast power coordination and DC-link stability are considered important^47,48^. This is behavior, as seen in Fig. 16, which is consistent with transient responses observed in adaptive and real-time EMS strategies^47,48^, and hybrid energy storage systems like battery-supercapacitor systems have been suggested in the literature to reduce such effects^44–46^. The current work is, however, centered on a battery-based system to test system-level energy management and control performance under representative operating conditions. The integration of supercapacitors and the assessment of their impact on transient current mitigation and battery lifetime are considered part of future research directions. In general, the findings reveal that the suggested EMS framework is effective to control the battery SOC and organize the power exchange in dynamic operating conditions to ensure the stability of the system-level performance and justify the relevance of the proposed method to the hybrid marine propulsion systems.

**Fig. 17:**
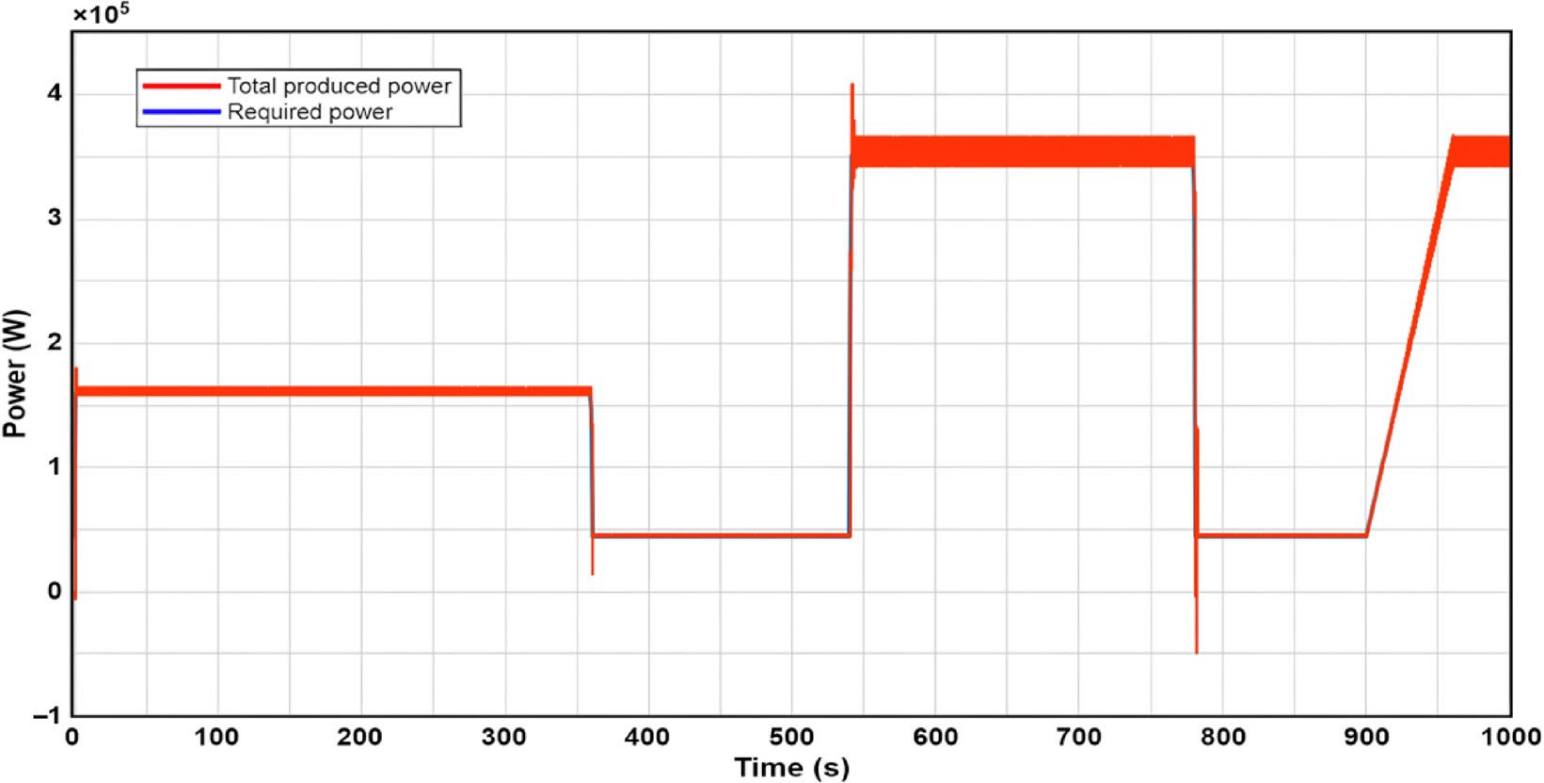
Comparing the required and total produced power.

Figure 17 shows the total generated power from the diesel generator and the battery compared to the total propulsion power demand over the 1000-s voyage. The generated power closely follows the propulsion load over the entire operating range, ensuring proper and stable coordination between the energy sources. During the first cruising period (0–360 s), the average power reaches a steady value of 1.6 × 10^5^ W, corresponding to the propulsion demand within an error of about 2%. When the load is reduced (≈ 380–500 s), the total output also decreases to approximately 0.6 × 10^5^ W, and under high-load conditions (≈ 540–780 s), the total generated power rises to almost 3.8 × 10^5^, with a deviation of less than 3%. In the last acceleration process (900–1000 s), the proposed EMS achieves the power balance in less than 2 s, which indicates the rapid dynamic compensation and smooth coordination between the diesel generator and the battery subsystem. Quantitatively, the maximum total power tracking error is within ± 3%, which is indicative of consistent real-time load sharing performance and stable DC bus regulation. Geertsma et al.^31^ and Balsamo et al.^32^ found similar accuracy, observing system power deviations of ± 4–5% under similar transient marine propulsion conditions. In total, the results shown in Fig. 17 indicate that the proposed SOC droop energy management strategy offers fast dynamic response, effective power balancing, and satisfactory DC bus stability, with performance similar to state-of-the-art hybrid marine propulsion systems.

**Fig. 18:**
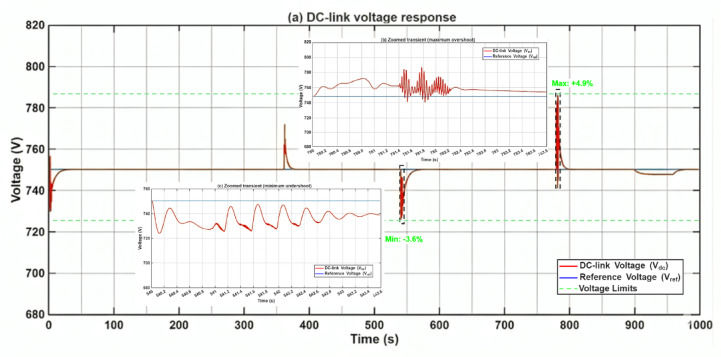
DC-link voltage response and transient performance: (**a**) Full time-domain response; (**b**) Zoomed view of the maximum voltage overshoot; (**c**) Zoomed view of the minimum voltage undershoot.

Figure 18 shows the DC-link voltage response with respect to its nominal reference value over the 1000-s voyage. Subfigure (a) presents the full time-domain response over the entire simulation period. Overall, the DC-link voltage is regulated around the nominal level of 750 V for most of the operating interval, confirming stable DC-bus operation and effective coordination between the generator and battery subsystems. During the initial operating phase, the voltage starts at approximately 730 V and reaches the nominal value of 750 V at around 25 s, indicating a short settling period and fast controller response. At approximately 360 s, a positive voltage excursion is observed, where the voltage rises to about 770 V before rapidly returning to the nominal value at around 365 s. A more pronounced undershoot occurs at approximately 540 s, where the voltage drops to about 723 V before recovering to 750 V at around 555 s. Between 780 s and 790 s, the voltage first decreases to approximately 740 V and then exhibits a sharp overshoot reaching about 787 V, after which it returns to the nominal value at around 800 s. During the final operating stage, only small deviations are observed, where the voltage drops slightly to about 745 V at around 900 s before stabilizing again at 750 V by approximately 970 s, indicating stable steady-state regulation after the major load transitions.

Subfigure (b) provides a zoomed view of the transient response under high dynamic loading conditions (≈ 781 s), highlighting the maximum voltage overshoot. The DC-link voltage exhibits oscillatory transient behavior before reaching a peak value of approximately 787 V, corresponding to a deviation of about + 4.9% relative to the nominal voltage. The voltage then gradually settles back toward the reference value, indicating a damped transient response and effective voltage regulation under dynamic conditions.

Subfigure (c) illustrates the transient response during the minimum voltage undershoot (≈ 540 s). The DC-link voltage drops to approximately 723 V, corresponding to a deviation of about − 3.6% relative to the nominal value. This undershoot is followed by a damped oscillatory recovery, where the voltage gradually returns to the reference level. This behavior reflects the system response to a rapid load disturbance and demonstrates effective damping and stable voltage regulation under dynamic operating conditions.

These deviations occur during rapid load changes and represent the inherent transient response of the DC-link under dynamic power exchange between the diesel generator and the battery. However, the voltage quickly returns to its nominal value within a short duration, indicating effective damping and fast recovery. Quantitatively, the voltage remains tightly regulated during steady-state operation, with transient deviations limited to short intervals during load disturbances. Compared with existing hybrid propulsion studies, similar DC-link voltage regulation ranges and transient behaviors have been reported. Geertsma et al.^31^ and Balsamo et al.^32^ observed DC-bus voltage variations typically within ± 3–5% under dynamic marine propulsion conditions, which is consistent with the transient deviations obtained in the present study. Overall, the results presented in Fig. 18 demonstrate that the proposed SOC-droop energy management strategy achieves stable voltage regulation, effective transient performance, and reliable system-level operation under the considered operating scenario.

Figures 8, 9, 10 and 11 show a close agreement between the reference and measured values of propulsion speed, torque, and dq-axis currents, which is in accordance with the control-oriented simulation scope of the present study and demonstrate the accuracy and stability of the proposed control architecture. These findings (Figs. 12, 13, 14, 15, 16 and 17) are further validated at the system level where the suggested SOC droop control and coordinated generator-battery management strategies are demonstrated to co-operatively achieve stable voltage regulation, equal power sharing, and stable energy distribution. These results, combined, indicate the effectiveness and steady behavior of the proposed control architecture in the considered representative operating scenario, which forms an initial foundation of future multi-scenario robustness assessment instead of an overall validation of such an architecture under all operating conditions.

These findings show better stability and efficient power coordination within the conditions of operation, especially in decreased DC-link voltage variation, reduced battery stress, and reduced transient oscillation. To further quantify these improvements, Table 7 presents a literature-based comparison of key dynamic performance indicators, including tracking accuracy, overshoot, and settling time, showing that the proposed control framework achieves a lower tracking error (≈ 0.7% compared to 2.3–4.7%), reduced overshoot, and faster settling time (≈ 2 s compared to 3–5 s) under the considered operating conditions.

**Table 7:** Literature-based quantitative comparison of representative dynamic performance indicators.

Study	System/application	Control method	Speed tracking error	Torque tracking error	Speed overshoot		Torque overshoot	Settling time
Geertsma et al^31^.	Hybrid marine propulsion architectures	Review of propulsion control strategies	2.3–4.7%	N/A		≈ 3%	N/A	N/A
Balsamo et al^32^.	Hybrid marine energy storage system	Optimization-based EMS	≈ 3.1%	N/A		≈ 2.0%	N/A	3–5 s
Zhang et al^41^.	Hybrid ship PMSM propulsion system	Adaptive finite-time command-filtered backstepping	N/A	≈ 1.9%		N/A	0%	2.8–3.2 s
This work	500-kW hybrid ferry propulsion system	SOC-droop EMS + PI–MTPA control	≈ 0.7%	< 2%		< 1.5%	< 3%	≈ 2 s (speed), 2–3 s (torque)

To further verify the reliability of the proposed SOC droop hybrid control strategy, a sensitivity analysis was carried out. In this experiment, the basic electrical parameters of the SRPM machine (Rs, Ld, and Lq) were perturbed by ± 5%, and a Gaussian perturbation of amplitude 1–2% was superimposed on the current and rotor speed measurement signals in a Simulink framework. Under nominal operating conditions, transient DC-link voltage deviations of + 4.9% and − 3.6% are observed. Under parameter perturbations, the DC-link voltage variation remains within approximately ± 3.5% of the nominal value, indicating that the control strategy maintains stable and well-regulated DC-link voltage even in the presence of parameter uncertainties and measurement noise. The propulsion speed tracking error increased slightly from approximately 0.7% to 1.1%, while torque tracking error remained below approximately 2.5%. Deviations in the d-axis and q-axis currents were limited to 1.2% and 2.3%, respectively. The aggregate generated power followed load fluctuations with a maximum mismatch below 3.5%, and the battery SOC was maintained within the 50 ± 1.2% band, ensuring safe operation within the 20–80% envelope. Overall, these results indicate that the proposed system maintains voltage stability, accurate torque/speed tracking, and balanced power sharing under realistic parameter uncertainties and sensor noise, demonstrating the robustness and practical applicability of the proposed control framework.

## Conclusions

This study presents and evaluates a simulation-based model for a 500 kW hybrid ferry propulsion system with a diesel generator and a lithium-ion battery within a flexible MATLAB/Simulink framework, designed to reflect representative operational conditions typical of Saudi Arabian hybrid vessels. The model includes an SRPM machine, advanced power converters, and hierarchical control strategies coordinating the generator, battery, and propulsion subsystems. Quantitatively, the proposed system demonstrates stable dynamic and steady-state performance under the operating scenario considered. For propulsion, speed tracking errors are below ≈ 0.7%, torque tracking errors are below ≈ 2%. The DC-link voltage exhibits transient deviations of + 4.9% and − 3.6% during dynamic conditions, while remaining more tightly regulated during steady-state operation. In addition, d-axis and q-axis current tracking errors are kept below 1% and 2%, respectively, which supports consistent torque generation and low electrical losses. Generator and battery powers are dynamically balanced to meet the propulsion demand, with a total power tracking deviation below 3%, while the battery SOC varies by less than ± 1% and well within the safe operating window of 20–80%. Overall, these results demonstrate stable operation, effective energy management, and accurate power sharing. The proposed SOC-droop strategy provides effective energy management with stable performance and real-time implementation capability compared to computationally intensive model predictive or adaptive control schemes. The model maintains DC-bus voltage around 750 V with limited transient deviations, demonstrating the stable behavior of the integrated control architecture under simulation conditions.

Ultimately, this work provides a simulation-based modular framework for hybrid propulsion system analysis aligned with Saudi Arabia’s Vision 2030 energy transition direction. The proposed model enables system-level evaluation of energy management and control strategies, and provides a foundation for future studies incorporating fuel consumption and emission analysis. The developed model is easily expandable to more operating conditions (e.g. different sea states, speed profiles and payload levels) and extendable in future studies to include hydrogen fuel cells and renewable energy sources, to enable potential emission reduction analysis and extended simulation-based evaluation. To summarize, the proposed hybrid propulsion framework is an effective way to incorporate the modelling, control, and performance evaluation of a proposed system in a single platform. The results of this research highlight the potential applicability and scalability in sustainable maritime operations, providing a foundation for future high-fidelity and experimental validation toward practical implementation.

Although the proposed framework demonstrates stable and effective performance in simulation, a clear gap still exists between the developed MATLAB/Simulink model and real-world ship propulsion systems. Real maritime propulsion systems are influenced by additional factors such as hydrodynamic disturbances, wave-induced loads, wind resistance, actuator delays, sensor imperfections, and mechanical drivetrain dynamics, which are not fully represented in the present simulation framework. Therefore, although the proposed model provides a reliable platform for control design and performance evaluation, further validation through high-fidelity vessel dynamics modeling, hardware-in-the-loop testing, and experimental implementation will be necessary to fully bridge the gap between simulation and real-world hybrid ship propulsion systems.

## Limitations and future work

The present validation is based on a single representative duty cycle and does not include route-level variability, environmental disturbances, payload sensitivity, or statistical scenario-based analysis. The present study does not include a detailed fuel consumption model, emission analysis, or quantitative generator stress metrics. Therefore, the reported improvements are based on power flow behavior and control performance rather than direct fuel or emission measurements. While the current research demonstrates the feasibility and satisfactory performance of a 500 kW hybrid ferry propulsion system, it is a simulation-based evaluation that is performed in the environment of the MATLAB/Simulink software. The 500 kW model covers a complete representative duty cycle but is based on assumptions of calm water, simplified environmental disturbances and only one vessel configuration of 500 kW. Future research will address these limitations by (i) using multi-scenario evaluation across a variety of sea states, weather conditions, and payload levels; (ii) using records of load profiles or synthetic load profiles with realistic noise and marine disturbances; (iii) combining the supplementary renewable energy resources such as hydrogen fuel cells and photovoltaic modules; (iv) comparing the SOC-droop strategy with alternative EMS techniques; (v) extrapolating the model for larger or different vessel types; (vi) using nonlinear flux linkage maps to better represent the magnetic saturation effects and evaluate their effects on torque ripple and performance; (vii) incorporating advanced battery modeling approaches that account for aging mechanisms and the impact of high transient current profiles on battery lifetime, as well as investigating hybrid energy storage configurations (e.g., battery–supercapacitor systems) to mitigate transient current peaks and reduce electrochemical stress under dynamic operating conditions; and (viii) incorporating ship motion dynamics, wave disturbances, and wind resistance models, as well as hardware-in-the-loop (HIL) testing to further validate the proposed control framework under realistic maritime operating conditions. Such enhancements will further increase the fidelity of the model for real-world applications, expand its usability, and support the evaluation of hybrid propulsion strategies aligned with Saudi Arabia’s long-term maritime energy transition objectives, while enabling a structured progression toward experimental and field-level validation.

## Data Availability

The datasets used and analyzed in this study are available from the corresponding author on reasonable request.
